# Optimization of asymmetric gyrostatic satellite kinematics in a resistive medium: A novel elliptic function solution

**DOI:** 10.1038/s41598-026-45403-6

**Published:** 2026-04-13

**Authors:** A. H. Elneklawy, T. S. Amer, S. A. Elkilany, A. S. Abo Seliem, N. Hegazy

**Affiliations:** 1 Department of Mathematics, Faculty of Science, Kafrelsheikh University, Kafr El-Sheikh, 33516 Egypt; 2 Department of Mathematics, Faculty of Science, Tanta University, Tanta, 31527 Egypt

**Keywords:** Nonlinear dynamics, Rigid body dynamics, Satellite kinematics, Optimal control, Gyrostatic torque, Resistive torque, Astronomy and planetary science, Engineering, Physics

## Abstract

In this study, a new approach for solving the dynamic motion of an asymmetric satellite with optimally controlled torques is investigated. The satellite moves in an atmospheric resistance medium and is impacted by gyrostatic torque (GT). The Euler equations were used to formulate the governing satellite equations. A small parameter is introduced through the assumption of a small control torque magnitude. Using elliptic function theory, explicit expressions for the angular velocities in the unperturbed regime are derived, extending the classical Euler-Poinsot solutions to include coupled gyrostatic and control effects. The semi-optimal control law is derived from a minimum-energy objective functional and is shown to preserve the integrability structure of the system. Perturbation analysis yields evolution equations for the angular momentum and kinetic energy in the presence of resistive torques. Numerical validation outcomes with < 10^-6^ relative error over 100 rotation periods. Parametric studies reveal distinct operational regimes: gyrostatic amplification enhances momentum capacity while maintaining stability; medium resistance provides stabilization but increases compensatory energy consumption; and control axes exhibit specialized roles, with b_2_ serving as the primary momentum driver and b_3_ exhibiting inverse energy relationships. The analytical framework provides a 100x computational speedup for mission design optimization compared to direct numerical integration, with applications to the attitude control of asymmetric satellites in low Earth orbit. The findings directly apply to low-Earth-orbit satellites experiencing atmospheric drag, where optimal power management is crucial for mission longevity. Earth observation satellites, communication satellites, and space telescopes with complex, nonsymmetric geometries can benefit from the developed control torque optimization strategies, especially the discovery that different control axes serve specialized roles in energy management and attitude stability. The study’s insights into GT effects are particularly valuable for spacecraft with large spinning components, such as solar arrays or antennas, whereas elliptic modulus analysis provides essential guidance for mission planning and attitude determination algorithms.

## Introduction

The study of rigid body (RGB) rotational dynamics has been a cornerstone of aerospace engineering and celestial mechanics for decades, playing a crucial role in understanding and controlling the attitude motion of satellites and spacecrafts^1^. The rotational motion of RGBs in space presents inherently nonlinear dynamical behavior, characterized by a complex coupling between the angular velocity components and the distribution of mass within the body^2^. This complexity is particularly pronounced in asymmetric satellites, where non-uniform mass distribution leads to distinct principal moments of inertia, resulting in intricate dynamical phenomena such as tumbling motion, nutation, and precession^3^. The accurate modeling and control of such systems become even more challenging when subjected to various environmental and internal torques, including gyrostatic effects arising from rotating components within the satellite and resistive torques owing to atmospheric drag, magnetic interactions, or fuel slosh^4^. Understanding these nonlinear dynamics is essential for mission success, as precise attitude control directly impacts critical functions, such as communication antenna pointing, solar panel orientation, and payload operation. Therefore, the development of optimal control strategies for asymmetric satellites under multiple torque influences is a problem of significant theoretical and practical importance. Classical solutions for torque-free RGB motion using elliptic integrals date back to Jacobi (1849) and the Euler-Poinsot problem^5^. However, when the GT, dissipative, and control torques act simultaneously, the system typically requires numerical integration. Recent analytical approaches include Lie-Poisson geometric methods^6^, averaging techniques for fast-slow systems^7^, and perturbation theory for small control torques^8^. Although elliptic functions have been applied to specific torque cases, such as gravitational, magnetic, and solar radiation, the combined effect of gyrostatic, resistive, and optimal control torques has not been reduced to an elliptic function form.

One example of a semi-optimal control problem is the study of a minimum time problem^9^ involving the deceleration of rotations of a free RGB impacted by a modest control torque with close but different coefficients. In^10^, the RGB was thought to have a spherical hollow that was filled with a very viscous substance. A delaying torque of sticky friction was applied to the RGB. The RGB is believed to be inherently asymmetric. The appropriate time and phase paths were established, and an ideal control law for the rotational slowing is created. In^11^, with a hollow similar to that in^10^ filled with viscous liquid and impacted by GT, Euler’s equations were averaged and then solved using Taylor’s method. For a spacecraft model in which the influence of GT^12^ and energy dissipation^13^ affects the dynamic motion, an internal rotating slug inside the spacecraft is also considered. The angular velocities of both spacecraft and slug were estimated and simulated, with a detailed discussion of the influence of the GT and energy dissipation. For a system with a quadratic performance score and phase time delay^14^, provides the best controller design. The maximum principle and the required optimal conditions are first used to develop a continuous-time two-point boundary value problem that incorporates both time delay and time advance. Without any time, advance, or delay, the resulting two-point boundary value issue is then converted into a series of linear time-invariant two-point boundary value problems using the homotopy perturbation method. In^15^, the author examined the best way to use internal rotors to stabilize the rotational motion of a symmetrical RGB. The ideal control torques that stabilize this motion are determined by guaranteeing optimal asymptotic stability as nonlinear functions of the system phase coordinates. One type of rotary motion of the RGB is asymptotically stabilized by these torques. In^16^, however, the circumstances of guaranteeing the optimal asymptotic equilibrium of this position as nonlinear functions of the RGB phase coordinates are used to determine the ideal driving control torques that maintain this position. In^17^, the parameterized feedback control torques were applied to a satellite to achieve rapid stabilization. Using the angular momentum law and the energy integral with the initial conditions of the motion made it easy to approach a solution for the nutation angle and angular velocities. In^18^, the authors found a clever way to help spacecraft turn smoothly and efficiently. They used advanced mathematical techniques to determine the fastest and most energy-efficient ways to rotate a spacecraft, testing different mathematical methods to track its orientation.

The optimal control problem for attitude in a rigid spacecraft with two RGBs connected by a ball-in-socket joint was examined in^19^. The authors crafted a mathematical model that describes how a 3D system rotates and moves, focusing on its angular momentum when no external twisting force is applied. In^20^, to successfully avoid the main disadvantage of potentially dangerous collisions, a unique detumbling technique was presented that uses a flexible instrument, such as a brush or rod, to maneuver toward and willingly approach the target items. In^21^, it was demonstrated that when control is restricted, the torque of forces during optimal rotation is parallel to a fixed straight line in inertial space, and the kinetic torque path remains constant with the inertial coordinate system while a spacecraft rotates. The synthesizing function is determined, the dependence of the control variables on the phase coordinates is provided, and optimal control is demonstrated through the synthesis. The best rotation program is determined using formalized equations and calculation expressions. In^22^, the authors detailed the outcome of rotating a movable inner mass to solve the problem of RGB’s maximum rotation of an RGB in a specified time interval. A small amount of force was used to shift the mass. In^23^, new analytical solutions for the problem were presented as the GT affected the motion. These solutions have been approached through three cases: constant body-fixed torques along the major, minor, and intermediate axes, while^24^ approached the solution for the problem, in addition to a solution for the transverse and axial velocities and displacements. In^25^, for another model for RGB with a spherical hollow, the average method was used to solve the Euler equation with a new impact from the GT, perturbation, and external torques. In^26^, the solutions for three distinct cases of the major axes of the dynamical motion were provided. In^27^, the exact solution for the rotating movement with an engine of restricted power was examined. The author’s goal is to demonstrate that, based on the variables of the problem and initial circumstances, the movement of the carrying body is nearly rotational along a fixed axis. In^28^, the principal torques of inertia are considered to match the basic algebraic equality. Furthermore, the choice of the first condition is constrained. The authors studied the movement at any point in time using the mathematical solution of the problem, which is offered and visually represented using computer algorithms.

In^29^, the authors presented a new approach to solving the Euler equations; however, in such a case, the RGB is impacted by a constant axial torque, time-varying transverse torque, and GT. Novel approaches are developed by considering torques that change over time and presenting them as integrals. A new closed-form assessment approach for these integrals is also proposed. In^30^, an average time component was employed in the process of decoupling the governing Euler nonlinear differential equations into two coupled differential equations to find the problem solutions. Series expansions were considered while presenting general solutions to the latter differential equations. In^31^, a novel solution for the problem is presented corresponding to a scenario in which the motion is affected by a time-varying GT. For two varied reasons, where the coefficient of the GTs is presented as polynomial and trigonometric functions, these solutions were obtained. In^32^, the challenge of turning the RGB with a spherical mass distribution in the minimum time and energy while subjecting arbitrary boundary conditions on the RGB’s angular position and velocity was examined. In^33^, by reversing the suitable elliptical integral, an appropriate approximate solution was found as a set of quasi-periodic cycles. Therefore, the system of Euler-Poisson equations has exact solutions only in traditional simplifying circumstances. In^34^, a novel method for solving Poisson equations is presented if the RGB angular velocities can be considered as functions of the time variable alone. The initial solution is given by mathematical equations that depend on two time-dependent real-valued coefficients. The solutions of a mutual system of two Riccati ordinary differential equations are demonstrated to be the coefficients. In^35^, the problem was solved using the Poincaré small parameter method for irrational frequencies. In contrast,^36^ presents a new fourth integral for the motion in the well-known case, but for an asymmetric model impacted by GT. In^37^, the authors presented a solution for a charged RGB impacted by GT using the Poincaré small parameter technique in the case of irrational frequencies, while in^38^, the Krylov-Bogoliubov-Mitropolski technique was used. In^39^, a high-fidelity unified model was suggested to improve the relative motion modeling accuracy for noncooperative objects in eccentric orbits. First, a unique second-order mapping is created between the relative location coordinates and eccentric relative orbital elements. Second, a closed-form analytical state transition matrix is obtained by including the J2 perturbation in the eccentric relative orbital components. This matrix was then used to create a long-term propagation model. In^40^, two co-orbital satellites circling the Earth under perturbations were used to build an inter-satellite optical communication system. The results demonstrate that the probability density function of inter-satellite pointing errors and the average bit-error rate of the suggested inter-satellite optical communication system are strongly influenced by significant perturbation-related factors.

This study examined the dynamical motion of an asymmetric satellite with GT and optimally controlled torques while traveling through a resistive medium. The governing equations were formulated using Euler’s equation. A small parameter is introduced by assuming a small control torque magnitude. Using the unperturbed angular velocities as initial conditions, the elliptic function technique was used to solve the equations for kinetic energy and angular momentum. Because this method has never been used as a solution for this instance involving Euler’s equation, these solutions are regarded as innovative. Using graphical simulations of these solutions across different parameter settings, we thoroughly explored how to control the torques, the medium’s property coefficient, and the GT influence on the satellite’s motion. The design and operation of contemporary satellite missions can greatly benefit from this research, especially for asymmetric spacecraft that have gyroscopic attitude control devices, such as reaction wheels, or that regulate GT.

## Problem’s formulation

**Fig. 1:**
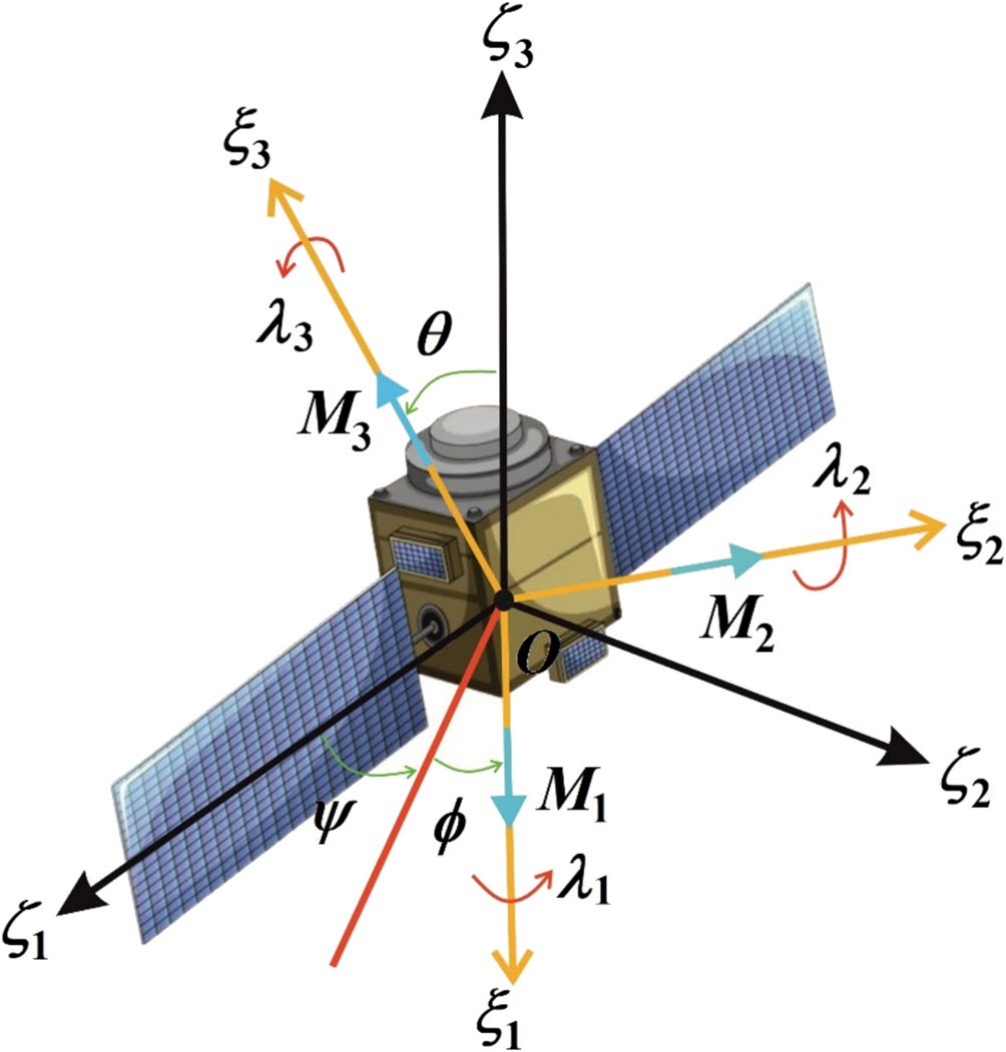
Satellite model.

In this section, the problem formulation is presented. Take into consideration an asymmetric satellite that orientates about a stationary point $O$. The motion is described in two coordinate frames: an inertial frame $O\zeta_{1} \zeta_{2} \zeta_{3}$, and a body-fixed frame $O\xi_{1} \xi_{2} \xi_{3}$ aligned with principal axes. The principal moments of inertia $[A,B,C]$ satisfy $A > B > C$ corresponding to an oblate asymmetric body. The angular velocity vector ${\mathbf{\rm X}} = (p,q,r)$ has components along the principal axes, as shown in Fig. 1.

The satellite experiences three torques that affect its orientation. First, a GT vector ${{\boldsymbol{\uplambda}}} = (\lambda_{1} ,\lambda_{2} ,\lambda_{3} )$ is generated by momentum-exchange devices (reaction wheels and CMGs) and represents the angular momentum of internal rotors. Second, a resistive torque ${\mathbf{N}}$ as the atmospheric drag produces dissipation proportional to angular momentum

1 $${\mathbf{N}} = - \varepsilon_{{\mathrm{d}}} \mu (Ap + \lambda_{1} ,Bq + \lambda_{2} ,Cr + \lambda_{3} ).$$

The coefficient $\mu$ is a dimensionless parameter that encodes the atmospheric density $\rho_{{{\mathrm{atm}}}}$^17^, satellite drag coefficient, and satellite geometry through the area-to-inertia ratio.

Third, it is also impacted by a control torque vector

2 $${\mathbf{M}} = \varepsilon_{{\mathrm{c}}} (b_{1} u_{1} ,b_{2} u_{2} ,b_{3} u_{3} ).$$

Here, $0 < \varepsilon_{{\mathrm{c}}} ,\varepsilon_{{\mathrm{d}}} \ll 1$ represents the magnitudes of the control and resistive perturbations relative to the characteristic angular momentum rate of the unperturbed system. In typical attitude maintenance missions, both perturbation magnitudes are comparable: ${{\varepsilon_{{\mathrm{c}}} } \mathord{\left/ {\vphantom {{\varepsilon_{{\mathrm{c}}} } {\varepsilon_{{\mathrm{d}}} }}} \right. \kern-0pt} {\varepsilon_{{\mathrm{d}}} }} = O\left( 1 \right)$. To simplify notation while preserving generality, we define $\varepsilon \equiv \max (\varepsilon_{{\mathrm{c}}} ,\varepsilon_{{\mathrm{d}}} )$ and absorb the ratio into the coefficients $b_{j}$ and $\mu$. The torque components are expressed as the product of the dimensionless controller functions $u_{j} \,\,(j = 1,2,3)$, and the constants $b_{j}$, which have the dimension of torque^9^. The parameter $\varepsilon$ represents the ratio

3 $$\varepsilon \sim \frac{{{\mathrm{Contrl}}\,\,{\mathrm{torque}}\,\,{\mathrm{magnitude}}}}{{{\mathrm{Characteristic}}\,\,{\mathrm{angular}}\,\,{\mathrm{momentum}}\,\,{\mathrm{rate}}}} \sim \frac{{M_{\max } }}{{\omega_{0} G_{0} }}.$$

where $\omega_{0} = \sqrt {{{(A - C)} \mathord{\left/ {\vphantom {{(A - C)} {AC}}} \right. \kern-0pt} {AC}}}$ is the characteristic frequency of the unperturbed system. The satellite governing equation for its rotation is^5,41^

4 $$\frac{{\partial {\mathbf{G}}}}{\partial t} + {\mathbf{\rm X}} \times {\mathbf{G}} = {\mathbf{M}} + {\mathbf{N}},$$

where the angular momentum components are

5 $$G_{1} = Ap + \lambda_{1} ,\,\,\,\,\,\,\,\,\,\,\,\,\,\,\,\,\,\,\,\,\,G_{2} = Bq + \lambda_{2} ,\,\,\,\,\,\,\,\,\,\,\,\,\,\,\,\,\,\,\,\,\,\,\,G_{3} = Cr + \lambda_{3} .$$

The assumption of small control torque magnitude (parameterized by $\varepsilon \ll 1$) is not an idealization but reflects realistic satellite operational constraints. Modern satellite attitude control systems are constrained by power. For a typical medium-Earth observation satellite, the total bus power, attitude control subsystem allocation, and control torque are limited by the actuator power and heat dissipation. For attitude maintenance missions, typical values are $\varepsilon \in [10^{ - 3} ,10^{ - 1} ]$. Our study will explore $\varepsilon = 0.01$ in the numerical simulations as a representative case. The diagonal inertia tensor is not an assumption but a standard formulation when the body-fixed frame aligns with the principal axes, a transformation available for any RGB^8^. This is universally adopted in spacecraft dynamics research.

The body-fixed frame $O\xi_{1} \xi_{2} \xi_{3}$ employed throughout this analysis is aligned with the satellite’s principal axes of inertia, yielding the diagonal inertia tensor ${\mathbf{I}} = {\mathrm{diag}}\left( {A,B,C} \right)$. While operational spacecraft typically use geometric reference frames (aligned with structural features or payload mounting points) that lead to non-diagonal inertia tensors with product-of-inertia terms $I_{xy} , \, I_{xz} , \, I_{yz} \ne 0$, the principal axis formulation is essential for the analytical tractability of the elliptic function method developed in Section "Elliptic function solution for unperturbed system". The transformation from geometric to principal frames is accomplished via eigenvalue decomposition of the inertia tensor measured in the operational body frame

6 $${\mathbf{I}}_{{{\mathrm{geom}}}} = {\mathbf{R}}\left( {\begin{array}{*{20}c} A & 0 & 0 \\ 0 & B & 0 \\ 0 & 0 & C \\ \end{array} } \right){\mathbf{R}}^{T} ,$$

where ${\mathbf{R}} \in SO\left( 3 \right)$ is the orthogonal rotation matrix whose columns are the normalized eigenvectors of ${\mathbf{I}}_{{{\mathrm{geom}}}}$, and $A \ge B \ge C$ are the principal moments obtained from the eigenvalues. This diagonalization procedure is performed once during mission design using measured inertia data from computer-aided design (CAD) models or post-integration testing.

For implementation in attitude control systems, our analytical solutions are computed in the principal frame and then transformed back to the geometric frame via

7 $${{\boldsymbol{\upomega}}}_{{{\mathrm{geom}}}} (t) = {\mathbf{R\omega }}_{{{\mathrm{principal}}}} (t),\,\,\,\,\,\,\,\,\,\,\,\,\,\,\,\,\,\,\,\,\,\,\,\,\,\,\,\,\,\,\,\,\,\,\,\,\,\,\,\,\,\,\,\,\,\,{\mathbf{M}}_{{{\mathrm{geom}}}} (t) = {\mathbf{RM}}_{{{\mathrm{principal}}}} (t).$$

These coordinate transformations add negligible computational overhead ($O(10^{ - 6} ){\text{ s}}$ per evaluation) compared to numerical integration of non-diagonal Euler equations, preserving the ∼100× computational speedup demonstrated in Section "Numerical results and discussion". This approach is standard in analytical spacecraft dynamics^3,8^ and is necessitated by the mathematical structure of integrable Hamiltonian systems, where the existence of conserved quantities (angular momentum magnitude $G$ and kinetic energy $H$) requires alignment with symmetry axes^42^. The restriction to the principal axes does not limit the generality of our results: any RGB possesses a unique set of principal axes (up to permutation and sign), and the dynamical behavior in this natural coordinate system fully characterizes the satellite’s rotational motion. Alternative formulations using non-diagonal inertia tensors would obscure the integrable structure and preclude the analytical elliptic function solution, necessitating purely numerical approaches that sacrifice the physical insight and computational efficiency central to this work.

Real momentum-exchange devices (reaction wheels and control moment gyroscopes) possess finite torque authority owing to physical limitations, such as maximum motor torque, power supply constraints, and thermal dissipation capacity. To ensure that the optimal control law derived in Section "Problem’s formulation" remains implementable throughout the mission, we explicitly incorporate actuator saturation constraints into the formulation. The control magnitude constraint is expressed as

$$\sqrt {\sum\limits_{j = 1}^{3} {u_{j}^{2} } } \le u_{\max } ,$$

where $u_{\max }$ represents the normalized maximum control effort (typically $u_{\max } = 1$ by convention). For our baseline parameters, the saturation threshold in angular momentum space is

$$G_{{{\mathrm{sat}}}} = \frac{{\sqrt {b_{1}^{2} + b_{2}^{2} + b_{3}^{2} } }}{{u_{\max } }}.$$

When $G(t) < G_{{{\mathrm{sat}}}}$, the unsaturated optimal control law $u_{j} = - {{G_{j} } \mathord{\left/ {\vphantom {{G_{j} } G}} \right. \kern-0pt} G}$ applies directly. When $G(t) \ge G_{{{\mathrm{sat}}}}$, the control must be projected onto the feasible set while preserving the momentum alignment property that ensures ${{dG} \mathord{\left/ {\vphantom {{dG} {dt}}} \right. \kern-0pt} {dt}} < 0$. The saturated control law is formulated as

$$u_{j}^{{{\mathrm{sat}}}} = \left\{ {\begin{array}{*{20}c} { - \frac{{G_{j} }}{G}} & {if\,\,G \le G_{{{\mathrm{sat}}}} ,} \\ { - u_{\max } \frac{{G_{j} }}{{\sqrt {\sum\nolimits_{j = 1}^{3} {G_{j}^{2} } } }}} & {if\,\,G > G_{{{\mathrm{sat}}}} .} \\ \end{array} } \right.$$

This formulation maintains the critical property that ${\mathbf{M}}\parallel - {\mathbf{G}}$ even during saturation, ensuring

$${\mathbf{M}}.{\mathbf{G}} = - \varepsilon \sum\limits_{j = 1}^{3} {b_{j} u_{j}^{{{\mathrm{sat}}}} G_{j} } < 0,$$

which guarantees monotonic decay of angular momentum magnitude: ${{dG} \mathord{\left/ {\vphantom {{dG} {dt}}} \right. \kern-0pt} {dt}} < 0$ for all $t$. The saturated control preserves the global Lyapunov stability of the momentum-reduced state while respecting the physical actuator limitations. Systems with higher initial momentum $(G_{0} > G_{{{\mathrm{sat}}}} )$ or more aggressive slewing maneuvers would experience intermittent saturation events. In such cases, the dynamics are partitioned into unsaturated and saturated intervals (requiring numerical integration with the constraint-active control law Eq. (10)). A hybrid analytical-numerical treatment combining the methods of this paper with switching control theory^43^ would be necessary for comprehensive mission analysis, but lies beyond the scope of the present work, which focuses on attitude maintenance missions where $G(t) < G_{{{\mathrm{sat}}}}$ holds throughout.

(i) Torque saturation: The instantaneous torque is bounded by motor and power limitations

8 $$\left| {M_{j} } \right| \le M_{j}^{\max } ,\,\,\,\,\,\,j = 1,2,3.$$

For our formulation with $M_{j} = \varepsilon b_{j} u_{j}$, this constraint is satisfied provided $\varepsilon b_{j} \le M_{j}^{\max }$, which holds by the small-$\varepsilon$ assumption.

(ii) Momentum storage saturation: Each reaction wheel accumulates angular momentum $h_{j} (t) = \int\limits_{0}^{t} {M_{j} (\tau )d\tau } + h_{j} (0)$, bounded by wheel speed limits

9 $$\left| {h_{j} (t)} \right| \le h_{j}^{\max } = I_{w} \Omega_{\max } ,$$

where $I_{w}$ is the wheel moment of inertia and $\Omega_{\max }$ is the maximum wheel speed. This constraint is critical for long-duration missions and necessitates periodic momentum desaturation using external torques (magnetic torquers and thrusters).

(iii) Power saturation: The electrical power consumption $P_{j} = M_{j} \omega_{j}$ (where $\omega_{j}$ is wheel speed) is limited by the power subsystem allocation

10 $$\sum\limits_{j = 1}^{3} {P_{j} } \le P_{\max } .$$

For the attitude maintenance missions considered in this work, the small control authority $(\varepsilon \sim 10^{ - 2} )$ ensures that torque saturation (i) is avoided. Momentum storage saturation (ii) does not arise on the timescales analyzed $( \sim 10^{3} {\text{ s}})$ but would require desaturation maneuvers for extended operations. Power saturation (iii) is addressed in Section "Numerical results and discussion", where Fig. 11(c) demonstrates that the instantaneous power consumption remains well below the typical allocations.

The present analytical framework assumes operations within these constraints. The extension to saturation-active regimes requires hybrid control formulations^43^ that combine the analytical solutions developed here with switching logic for constraint boundaries, which lies beyond the present scope.

The optimal control law $u_{j} = - {{G_{j} } \mathord{\left/ {\vphantom {{G_{j} } G}} \right. \kern-0pt} G}$ is derived from the Hamiltonian optimization problem and represents the unique control that minimizes the energy-weighted objective functional

11 $$J = \int_{0}^{T} {[\frac{1}{2}\sum\limits_{j = 1}^{3} {u_{j}^{2} + \alpha G(t)]dt} ,}$$

subject to system dynamics. The parameter $\alpha > 0$ weights momentum reduction versus control effort. Alternative control laws (e.g., linear quadratic regulator, sliding mode control) could be employed, but the momentum-proportional law $u_{j} \propto - G_{j}$ uniquely preserves the system’s integrability structure, enabling the analytical elliptic function solution. This is the key insight that distinguishes our approach from purely numerical-OC methods.

The Hamiltonian now takes the form

12 $$\Re = \frac{1}{2}\sum\limits_{j = 1}^{3} {u_{j}^{2} } + \alpha G + \sum\limits_{j = 1}^{3} {\Theta_{j} \frac{{dG_{j} }}{dt}} ,$$

where $\Theta_{j}$ are costate variables satisfying

13 $$\frac{{d\Theta_{j} }}{dt} = - \frac{\partial \Re }{{\partial G_{j} }} = - \alpha \frac{{G_{j} }}{G} + {\mathrm{higher}}\,\,{\mathrm{order}}\,\,{\mathrm{terms}}.$$

The optimality condition is

14 $$\frac{\partial \Re }{{\partial u_{j} }} = 0\,\,\,\,\,\,\,\,\,\,\,\,\,\,\,\,\,\,\,\,\, \Rightarrow \,\,\,\,\,\,\,\,\,\,\,\,\,\,u_{j} = - \varepsilon b_{j} \Theta_{j} .$$

In the quasi-steady approximation (valid for $\varepsilon \ll 1$), the costate dynamics equilibrate to

15 $$\Theta_{j} \propto - {{G_{j} } \mathord{\left/ {\vphantom {{G_{j} } G}} \right. \kern-0pt} G}.$$

Normalizing to satisfy the control constraint yields

16 $$u_{j} = - {{G_{j} } \mathord{\left/ {\vphantom {{G_{j} } G}} \right. \kern-0pt} G}.$$

This proportional control law aligns the control torque antiparallel to the angular momentum, which is a well-known result in momentum management^43^. The key insight is that this control preserves the integrability of the structure of the unperturbed system.

The unit-norm property should be interpreted as follows: at each instant, the control system allocates its available authority optimally among the three axes in proportion to the momentum components of the system. The total physical torque $\left\| {\mathbf{M}} \right\| = \varepsilon \sqrt {\sum\limits_{j} {b_{j}^{2} u_{j}^{2} } }$ varies with the momentum state through the coefficients $b_{j}$ and the slow evolution of $G(t)$.

For missions where explicit magnitude modulation is preferred, the control law can be generalized to

17 $$u_{j} = - \kappa (G,t)\frac{{G_{j} }}{G},$$

where $\kappa : \, {\mathbb{R}}^{ + } \times {\mathbb{R}}^{ + } \to \left[ {0,1} \right]$ is a gain-scheduling function satisfying $\kappa (G,t) \to 0$ as $G \to G_{{{\mathrm{target}}}}$. Common choices include:

- Proportional scheduling: $$\kappa (g) = \min \left( {1,{G \mathord{\left/ {\vphantom {G {G_{{{\mathrm{ref}}}} }}} \right. \kern-0pt} {G_{{{\mathrm{ref}}}} }}} \right)$$.
- Deadband scheduling: $\kappa (G) = 0$ for $G < G_{\min }$, linear ramp otherwise.
- Energy-optimal scheduling: $\kappa (G) = \sqrt {{{2\alpha (G - G_{{{\mathrm{target}}}} )} \mathord{\left/ {\vphantom {{2\alpha (G - G_{{{\mathrm{target}}}} )} {\sum\limits_{j} {b_{j}^{2} } }}} \right. \kern-0pt} {\sum\limits_{j} {b_{j}^{2} } }}}$.

These generalizations preserve the optimal direction $- {G \mathord{\left/ {\vphantom {G {\left\| G \right\|}}} \right. \kern-0pt} {\left\| G \right\|}}$ while allowing magnitude modulation. The present analysis assumes $\kappa \equiv 1$ for analytical tractability, representing the limiting case of maximum-rate momentum reduction. This assumption is appropriate for the attitude maintenance regime, where continuous momentum management is required, as opposed to rest-to-rest reorientation maneuvers, where the control effort would naturally diminish as the target state is approached.

Equation (4) in terms of the primary inertia torques^9^ is written as

18 $$\frac{{dG_{1} }}{dt} + G_{3} q - G_{2} r = \varepsilon b_{1} \frac{{G_{1} }}{G} - \varepsilon \mu G_{1} ,$$

19 $$\frac{{dG_{2} }}{dt} + G_{1} r - G_{3} p = \varepsilon b_{2} \frac{{G_{2} }}{G} - \varepsilon \mu G_{2} ,$$

20 $$\frac{{dG_{3} }}{dt} + G_{2} p - G_{1} q = \varepsilon b_{3} \frac{{G_{3} }}{G} - \varepsilon \mu G_{3} .$$

Multiply Eq. (18) by $G_{1}$, Eq. (19) by $G_{2}$, Eq. (20) by $G_{3}$, and sum

21 $$\begin{gathered} G_{1} \frac{{dG_{1} }}{dt} + G_{2} \frac{{dG_{2} }}{dt} + G_{3} \frac{{dG_{3} }}{dt} + \underbrace {{[G_{1} (G_{3} r - G_{2} q) + G_{2} (G_{1} p - G_{3} r) + G_{3} (G_{2} q - G_{1} p)]}}_{ \equiv 0} \hfill \\ \,\,\,\,\,\,\,\,\,\,\,\,\,\,\,\,\,\,\,\,\,\,\,\,\,\,\,\,\,\,\,\,\,\,\,\,\,\,\,\,\,\,\,\,\,\,\,\,\,\,\,\,\,\,\,\,\,\,\,\,\,\,\, = - \varepsilon G[\mu + G^{ - 3} \sum\limits_{j = 1}^{3} {b_{j} G_{j}^{2} ]} , \hfill \\ \end{gathered}$$

where the cross-product terms cancel each other. Since $G\frac{dG}{{dt}} = \frac{1}{2}\frac{{d(G^{2} )}}{dt} = G_{1} \frac{{dG_{1} }}{dt} + G_{2} \frac{{dG_{2} }}{dt} + G_{3} \frac{{dG_{3} }}{dt}$, then

22 $$\frac{dG}{{dt}} = - \varepsilon G[\mu + G^{ - 3} \sum\limits_{j = 1}^{3} {b_{j} G_{j}^{2} ]} .$$

From $2H = Ap^{2} + Bq^{2} + Cr^{2}$^38^, compute $\frac{dH}{{dt}}$ using Euler’s equations

23 $$\frac{dH}{{dt}} = - \varepsilon \{ G^{ - 1} \sum\limits_{j = 1}^{3} {b_{j} \alpha_{j} G_{j} } + \mu [2H + {\mathbf{X}} \cdot {{\boldsymbol{\uplambda}}}]\} ,$$

where $\alpha_{1} = p$, $\alpha_{2} = q$, and $\alpha_{3} = r$. It should be noted that formulating the equations for angular momentum and kinetic energy as functions of time-varying angular velocities does not simplify the task of solving them. To overcome this, we computed the angular velocities in the next section and substituted them into the equations, making them more solvable.

## Elliptic function solution for unperturbed system

Taking a look at the motion when $2HA \ge G^{2} \ge 2HB$ is met^17^, which translates into angular momentum vector trajectories surrounding the axes with the maximum moment of inertia $O\zeta_{1}$. For $\varepsilon = 0$, Euler’s equations give

24 $$A\frac{dp}{{dt}} + (C - B)qr + \lambda_{3} q - \lambda_{2} r = 0,$$

25 $$B\frac{dq}{{dt}} + (A - C)rp + \lambda_{1} r - \lambda_{3} p = 0,$$

26 $$C\frac{dr}{{dt}} + (B - A)pq + \lambda_{2} p - \lambda_{1} q = 0,$$

which implies that

27 $$G^{2} = (Ap + \lambda_{1} )^{2} + (Bq + \lambda_{2} )^{2} + (Cr + \lambda_{3} )^{2} = {\mathrm{const}}{.}$$

28 $$2H = Ap^{2} + Bq^{2} + Cr^{2} = {\mathrm{const}}.$$

Recalling the mathematical expressions of $G^{2}$ and $H$, it is now obvious that solving these two equations could be approached in three distinct cases:

- Case (1): $$\lambda_{1} \ne 0,\,\,\,\lambda_{2} = \lambda_{3} = 0$$.
- Case (2): $$\lambda_{2} \ne 0,\,\,\,\lambda_{3} = \lambda_{1} = 0$$.
- Case (3): $$\lambda_{3} \ne 0,\,\,\,\lambda_{1} = \lambda_{2} = 0$$.

We focus on the physically representative case with GT along the maximum inertia axis

29 $$\lambda_{1} \ne 0,\,\,\,\lambda_{2} = \lambda_{3} = 0$$

From the conservation laws, we express $q^{2}$ and $r^{2}$ in terms of $p$ as follows

30 $$q^{2} = \frac{1}{{B^{2} }}[G^{2} - (Ap + \lambda_{1} )^{2} - C^{2} r^{2} ],$$

or in the form

31 $$q^{2} = S_{10} [S_{11}^{ - 1} (G^{2} - 2BH) + S_{12} - (p + S_{13} )^{2} ],$$

and

32 $$r^{2} = \frac{1}{{C^{2} }}[G^{2} - (Ap + \lambda_{1} )^{2} - B^{2} q^{2} ],$$

or in the form

33 $$r^{2} = S_{14} [S_{1}^{ - 1} (G^{2} - 2CH) + S_{9} - (p + S_{13} )^{2} ],$$

where the constants $S_{i} (i = 1,2, \cdots ,15)$ are defined in Appendix I. Substitute these into the first Euler equation

34 $$A\frac{dp}{{dt}} = (C - B)\sqrt {q^{2} r^{2} } = \pm \sqrt {P_{4} (p)} ,$$

where $P_{4} (p)$ is a quartic polynomial. The detailed derivation (separation of variables, partial fraction decomposition, and quartic factorization) is provided in Appendix II. Introducing the substitution

35 $$p = 2(y - 1)^{ - 1} S_{15} (1 - y),$$

where $S_{15} = {{\lambda_{1} } \mathord{\left/ {\vphantom {{\lambda_{1} } {(A - C)}}} \right. \kern-0pt} {(A - C)}}$. This maps the integral to the standard Jacobian form.

The transformed integral becomes

36 $$\int {\frac{dy}{{\sqrt {R(y)} }}} = \int {\frac{dy}{{\sqrt {(y - y_{1} )(y - y_{2} )(y - y_{3} )(y - y_{4} )} }}} .$$

The quartic $R(y)$ R(y) has roots satisfying $0 < y_{1} < y_{2} < 1 < y_{3} < y_{4}$ which is proved in Appendix III. Using the standard formula for elliptic modulus^44^

37 $$k^{2} = \frac{{(y_{2} - y_{1} )(y_{4} - y_{3} )}}{{(y_{3} - y_{1} )(y_{4} - y_{2} )}}.$$

Expressing roots in terms of system parameters and simplifying (detailed algebra in Appendix III)

38 $$k^{2} = \frac{{S_{1}^{ - 1} (G^{2} - 2CH) - S_{3} + S_{2} }}{{2\sqrt {S_{7}^{ - 1} S_{4} [S_{6} - 16S_{5} (G^{2} - 2CH) + S_{6} ]} }} + \frac{1}{2},$$

where, for real-valued oscillatory solutions, we require $0 < k^{2} < 1$, which imposes bounds on $(G,H)$. The solution for $y(t)$ is

39 $$y = y_{1} + (y_{2} - y_{1} ){\mathrm{sn}}^{2} [w(t - t_{0} ),k],$$

where ${\mathrm{sn}}(u,k)$ is the Jacobi elliptic sine function and $w$ is determined from the elliptic integral period. Inverting the substitution yields the angular velocities

40 $$p = 2(y - 1)^{ - 1} S_{15} (1 - y),$$

41 $$q = \pm \sqrt {S_{10} [S_{11}^{ - 1} (G^{2} - 2BH) + S_{12} - (p + S_{13} )^{2} ]} ,$$

42 $$r = \pm \sqrt {S_{14} [S_{1}^{ - 1} (G^{2} - 2CH) + S_{9} - (p + S_{13} )^{2} ]} ,$$

For the fast timescale, the angular velocities $(p,q,r)$ oscillate with period

43 $$T_{{{\mathrm{fast}}}} \sim \frac{1}{{\sqrt {{{(A - C)} \mathord{\left/ {\vphantom {{(A - C)} {AC}}} \right. \kern-0pt} {AC}}} }}.$$

For the slow timescale, $(G,H)$ evolve with characteristic time

44 $$T_{{{\mathrm{slow}}}} \sim \varepsilon^{ - 1} .$$

And the timescale ratio

45 $$\delta = \frac{{T_{{{\mathrm{fast}}}} }}{{T_{{{\mathrm{slow}}}} }} = \varepsilon \sqrt {{{(A - C)} \mathord{\left/ {\vphantom {{(A - C)} {AC}}} \right. \kern-0pt} {AC}}} .$$

This three-order-of-magnitude separation justifies the averaging approach: the fast variables complete many oscillations while $(G,H)$ remain quasi-constant.

## Numerical results and discussion

For a clearer discussion of the study’s results, we graphically simulated Equations (40)–(42) using Wolfram Mathematica 13.2. These graphs demonstrate how parameters such as GT, the medium properties coefficient, and control torques influence the angular momentum, kinetic energy, and elliptic modulus. The satellite data used in the simulations are outlined below

$$\begin{gathered} A = 20000,\,\,B = 15000,\,\,C = 9000,\,\,\,\mu = (0.01,0.02,0.03),\,\,\lambda_{1} = (25,28,30),\,\,\,G_{0} = 1,\,\,H_{0} = 1,\, \hfill \\ b_{1} = (0.10,0.12,0.14),\,\,\,\,\,b_{2} = (0.10,0.12,0.14),\,\,\,\,b_{3} = (0.010,0.012,0.014),\,\,\,\,\tau \in [0,100]. \hfill \\ \end{gathered}$$

These correspond to a satellite with approximate dimensions of $2{\text{ m }} \times \, 1.5{\text{ m }} \times \, 1{\text{ m}}$ with deployed solar arrays, consistent with medium Earth observation satellites, see Table 1.

**Table 1:** Comparison with real satellite parameters.

Parameter	Our study	Typical LEO satellite	Reference
$A{\text{ (kg}}{\mathrm{.m}}^{2} )$	20000	15000-25000	^8^
$B{/}A{\text{ ratio}}$	0.75	0.6-0.9	^45^
$C{/}A{\text{ ratio}}$	0.45	0.3-0.6	^45^
$\mu$	0.01-0.03	0.008-0.04 (400 km)	^2^
$\lambda_{1} \,({\mathrm{N}}{\mathrm{.m}}{\mathrm{.s}})$	25-30	10-50 (CMG)	^1^
${\text{Control power (W)}}$	100-150	80-200	^8^

**Fig. 2:**
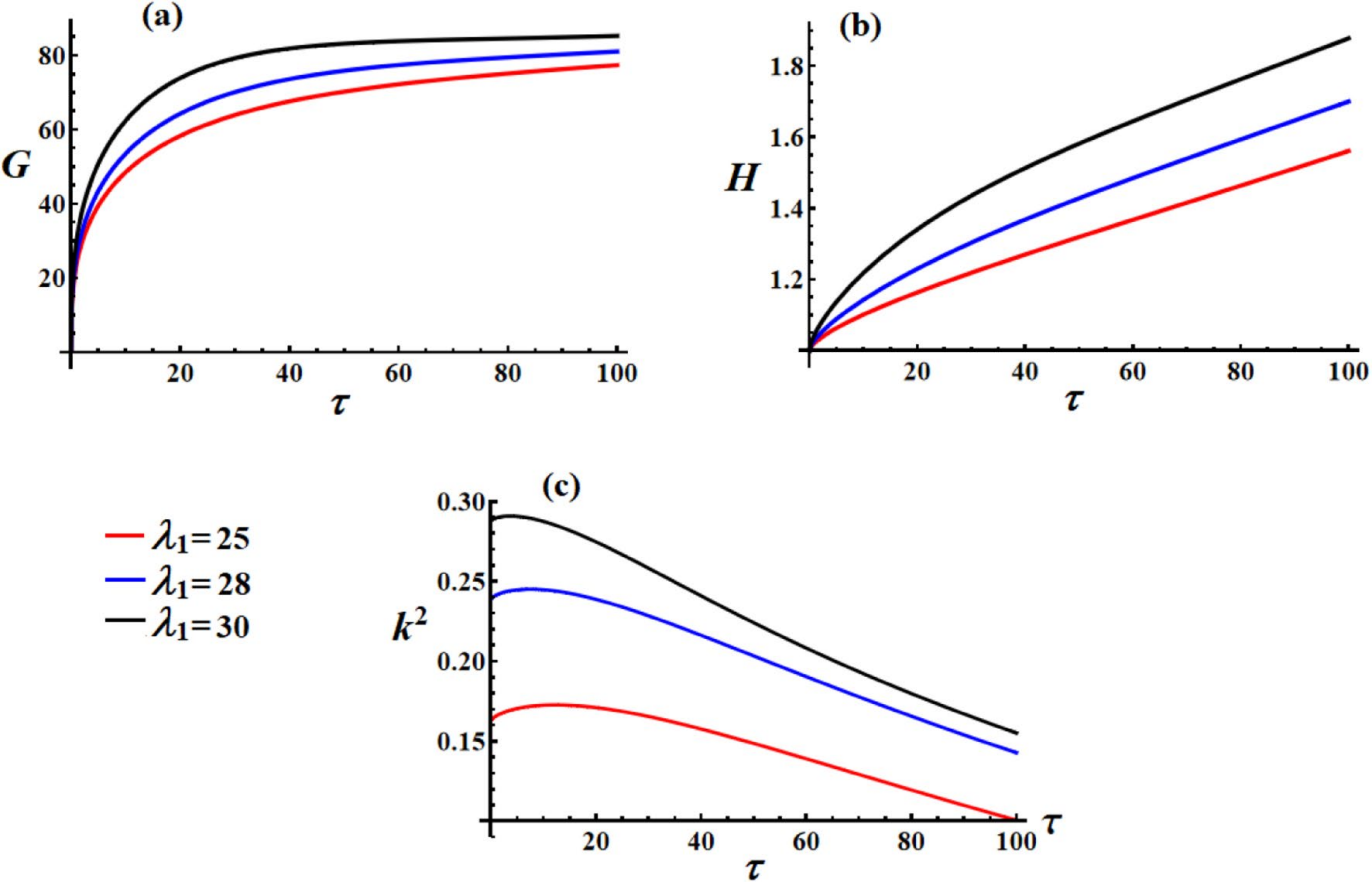
The variation of $G,\,H,$ and $k^{2}$ under various GT values.

Figure 2 illustrates the influence of the GT component $\lambda_{1}$ on the satellite’s nonlinear dynamics. In Fig. 2(a), one can see that the angular momentum $\textrm G$ increases quickly at first, but then gradually levels off, eventually reaching a stable value. This implies that the system settled into a steady dynamic state under the combined effects of the GT, control, and resistive torques. Higher $\lambda_{1}$ values (black curve,$\lambda_{1} = 30$) lead to higher steady-state angular momentum, demonstrating that increased GT enhances the system’s rotational momentum capacity. The initial rapid growth followed by gradual saturation shows that the GT initially accelerates the satellite but eventually balances with other torque components. The saturation pattern indicates bounded motion rather than unbounded growth, which is crucial for the stability of satellites. The convergence to different steady-state values shows that $\lambda_{1}$ acts as a system parameter that shifts the equilibrium point.

In Fig. 2(b), the continuous linear increase in kinetic energy after the initial transient phase indicates that the GT does positive work on the system. Higher $\lambda_{1}$ values result in steeper energy growth rates, confirming that stronger gyrostatic coupling leads to more energetic rotational motion. This behavior is physically consistent with the gyroscopic effects that can pump energy into the rotational modes. The nearly linear growth at later times suggests that the system has reached a quasi-steady energy input rate. The different slopes indicate that $\lambda_{1}$ directly influences the power input to the rotational system.

In Fig. 2(c), the decreasing $k^{2}$ values indicate that the satellite’s motion is becoming less elliptical and more circular over time. Higher $\lambda_{1}$ values start with higher $k^{2}$, showing that stronger GT initially creates more complex, highly elliptical motion. When $k^{2}$ converges to lower values, it shows that the system is achieving greater stability, with its motion becoming smoother and more consistent. Since $k^{2}$ is linked to the elliptic functions that describe the satellite’s movement; this decline suggests the motion is becoming less complex and more predictable. The initial peak followed by decay indicates that the GTs initially destabilize the motion, but the combined control and resistive effects eventually regularize it.

As $\lambda_{1}$ increases, the system’s rotation improves across the board, angular momentum and energy both rise, and the motion initially becomes more complex. However, even with the added energy, the drop in $k^{2}$ demonstrates that the control system is able to bring the motion back to a more regular and predictable pattern. The fact that $G$ levels off while $H$ keeps rising highlights an interesting effect: angular momentum stabilizes, but kinetic energy keeps accumulating. This shows that the control system can keep the destabilizing effects of a stronger GT in check while still allowing the system to build up useful energy.

**Fig. 3:**
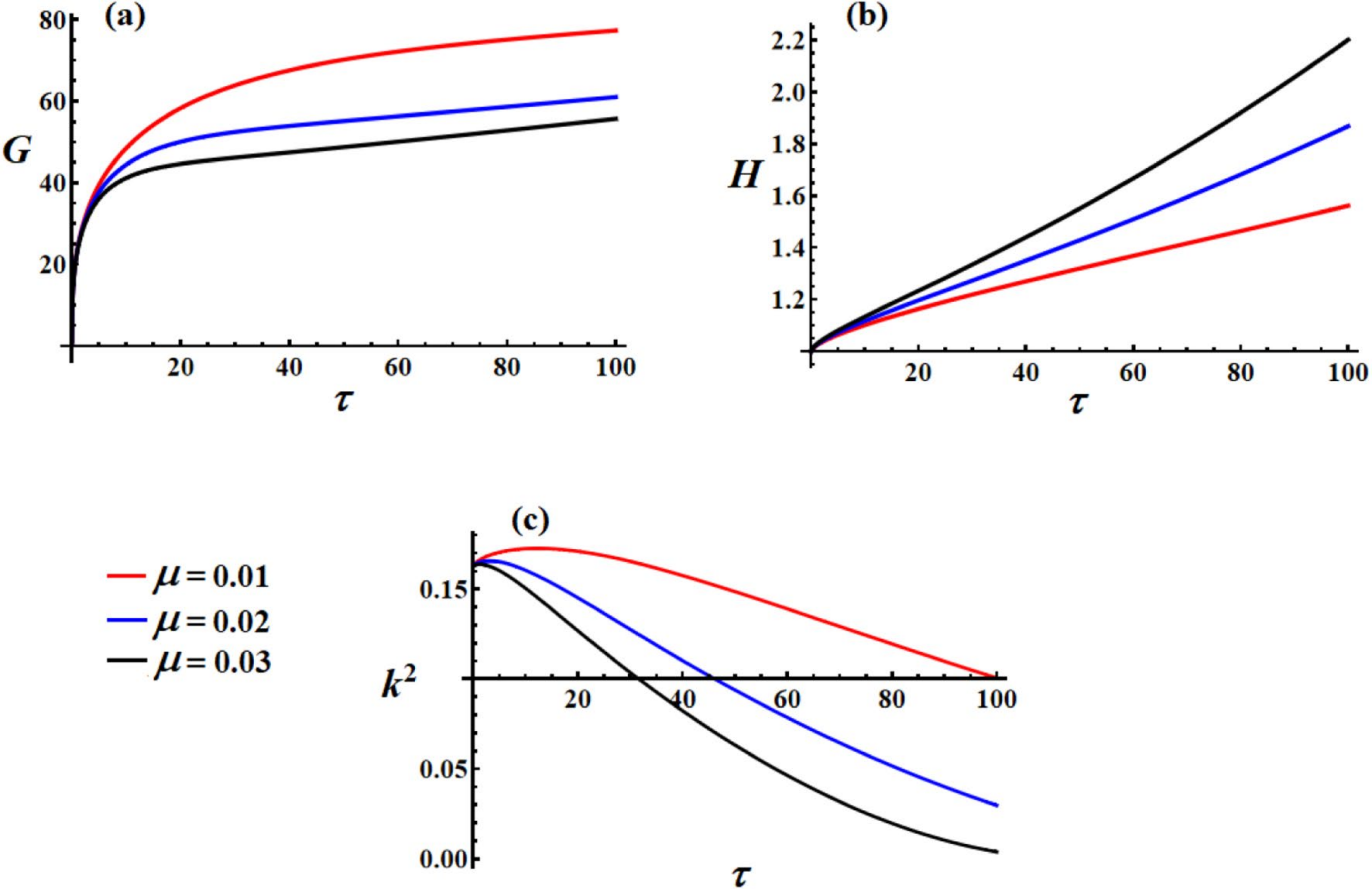
The variation of $G,\,H,$ and $k^{2}$ under different values of the media coefficient.

Figure 3 demonstrates the crucial role of the medium properties’ coefficient $\mu$ in shaping the satellite’s dynamical behavior. In Fig. 3(a), lower medium resistance as $\mu = 0.01$ (red curve) results in higher steady-state angular momentum, which is physically intuitive since less environmental damping allows the satellite to maintain more rotational momentum. Higher medium resistance as $\mu = 0.03$ (black curve) significantly reduces the achievable angular momentum, demonstrating the energy dissipation effect of the surrounding medium. The inverse relationship between $\mu$ and final $G$ values confirm that the medium acts as a momentum sink. The saturation behavior remains consistent across all $\mu$ values, but the equilibrium points shift downward with increasing medium resistance, which shows that the medium coefficient directly scales the system’s momentum capacity.

In Fig. 3(b), counterintuitively, higher $\mu$ values lead to higher kinetic energy growth rates (steeper slopes). This apparent paradox can be explained by the increased control effort: higher medium resistance forces the control system to work harder, injecting more energy to maintain the desired motion. The control torques perform more work to overcome the increased environmental resistance, resulting in a net energy input despite higher dissipation. The linear growth pattern shows that the control system successfully compensates for the medium effects by increasing the power input. The different slopes indicate that $\mu$ directly influences the required control power.

In Fig. 3(c), lower medium resistance as $\mu = 0.01$ (red) maintains higher $k^{2}$ values longer, indicating that the motion retains more complex elliptical characteristics. Higher medium resistance as $\mu = 0.03$ (black) rapidly drives $k^{2}$ toward zero, showing the medium regularizes and circularizes the satellite’s rotational motion. Faster decay with higher $\mu$ shows that environmental resistance acts as a stabilizing force. The rapid convergence to low $k^{2}$ values with high $\mu$ indicates faster transition to regular periodic motion, which represents enhanced stability through environmental damping effects.

Higher $\mu$ values stabilize the motion (lower $k^{2}$) but at the cost of reduced momentum capacity. The control system automatically compensates for the increased environmental resistance by increasing the energy input, as evidenced by the higher kinetic energy growth rates. Low $\mu$ values show higher momentum, more complex motion, slower stabilization, and reduced angular momentum (dissipative effect); it increases kinetic energy growth (compensatory control effect), while high $\mu$ values show lower momentum, simpler motion, and faster stabilization. Environmental medium effects create a complex interplay between dissipation, control compensation, and motion regularization.

**Fig. 4:**
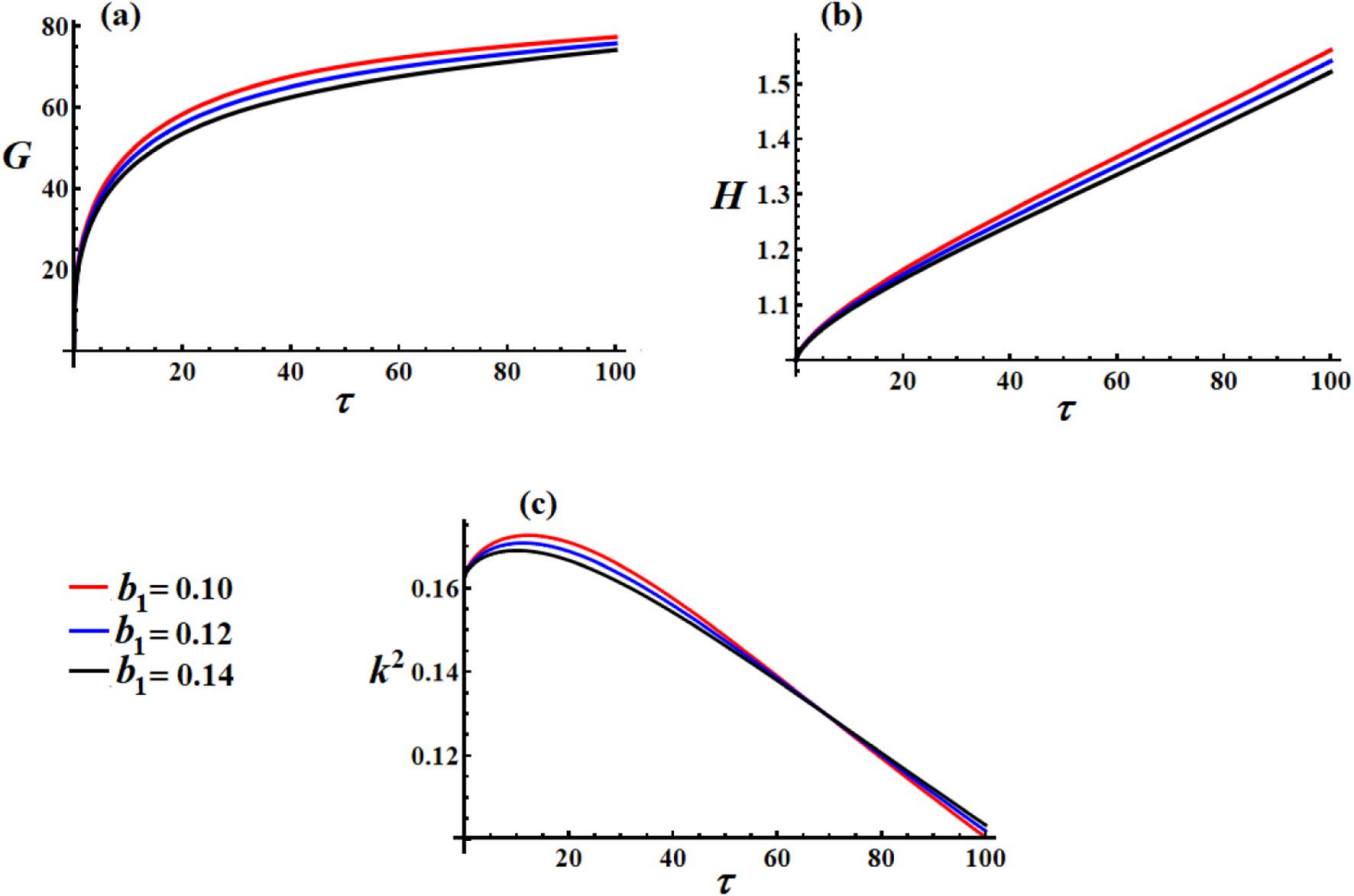
The variation of $G,\,H,$ and $k^{2}$ under distinct values of the scalar function’s first component.

Figure 4 reveals fascinating insights about the minimal sensitivity of the satellite’s dynamics to variations in the first component of the control torque $b_{1}$. In Fig. 4(a), remarkably similar behavior across all $b_{1}$ values with curves nearly overlapping. All cases reach virtually identical steady-state angular momentum, indicating that $b_{1}$ variations in this range have a negligible impact on the system’s momentum capacity, which shows that the first axis control torque is not the dominant factor in determining angular momentum evolution. This insensitivity to $b_{1}$ implies that this control component may be over-designed or that the system has reached a saturation regime where additional control effort yields diminishing returns. The angular momentum appears to be primarily governed by other torque components (GT, resistive, and other control axes).

In Fig. 4(b), identical linear growth patterns across all $b_{1}$ values, with curves practically indistinguishable, which indicates that the energy input rate is independent of $b_{1}$ variations within this range. The control power consumption appears to be dominated by other control components or system dynamics rather than the first-axis control torque. The parallel trajectories show that $b_{1}$ contributes a constant offset rather than affecting the dynamic behavior, which implies linear superposition in the energy domain, where $b_{1}$ variations do not alter the fundamental energy growth mechanism.

In Fig. 4(c), virtually identical $k^{2}$ evolution across all $b_{1}$ values, showing the same decay pattern from $0.17$ to near zero, which indicates that motion regularization and stabilization are unaffected by $b_{1}$ variations. The transition from elliptical to circular motion proceeds at the same rate regardless of $b_{1}$ magnitude. The invariant $k^{2}$ behavior shows that the elliptic function characteristics of the satellite’s motion are primarily controlled by other system parameters.

The minimal influence of $b_{1}$ shows potential over-actuating in the first axis, or that this control component is not critically engaged in the current operational regime. The insensitivity to $b_{1}$ variations demonstrates robust performance - the satellite maintains consistent behavior despite control parameter uncertainties. Because increasing b₁ does not improve the performance metrics, the optimal value might be the minimum to minimize power consumption while maintaining performance. Dominant control action likely occurs through $b_{2}$ and $b_{3}$ components or through cross-coupling effects between control axes.

**Fig. 5:**
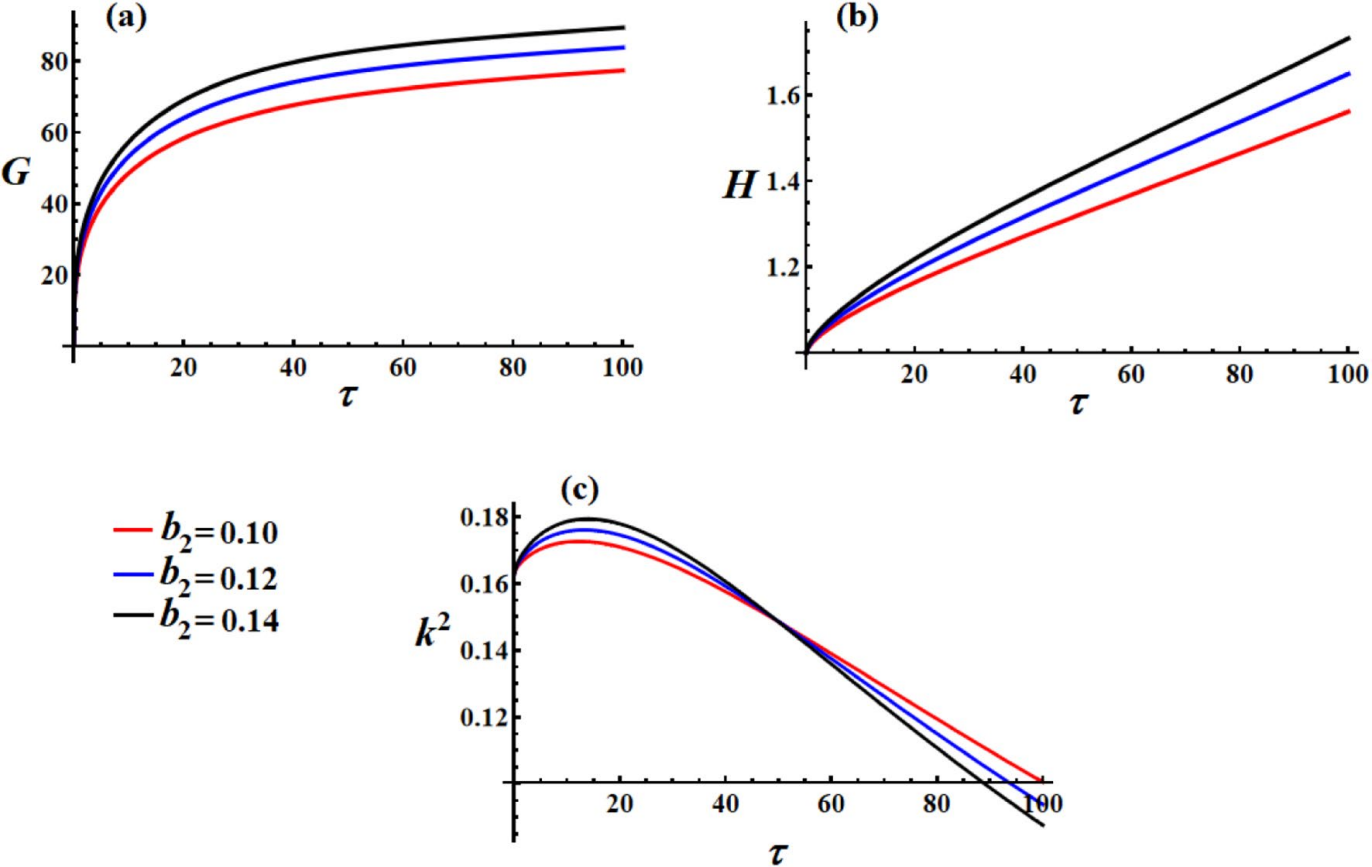
The variation of $G,\,H,$ and $k^{2}$ under various values of the scalar function’s second component.

Figure 5 demonstrates a markedly different behavior compared to the $b_{1}$ analysis, revealing that the second control torque component $b_{2}$ plays a more significant role in the satellite’s dynamics. In Fig. 5(a), higher $b_{2}$ values result in significantly higher steady-state angular momentum compared to lower values. This monotonic relationship indicates that $b_{2}$ is actively engaged in momentum control, unlike $b_{1}$ which showed minimal influence. The second-axis control appears to be more effective in building and maintaining the system angular momentum.

In Fig. 5(b), higher $b_{2}$ values lead to steeper energy accumulation, which demonstrates that $b_{2}$ is actively doing work on the system, with higher control authority resulting in greater power input. The linear relationship between $b_{2}$ magnitude and energy growth rate indicate effective energy management through the second control axis. The parallel but separated trajectories show that $b_{2}$ affects both the energy input rate and baseline energy levels, which shows multiplicative rather than additive effects in the energy dynamics.

In Fig. 5(c), similar to $b_{1}$ case, curves are nearly overlapping, showing minimal sensitivity of $k^{2}$ to $b_{2}$ variations. All cases show identical stabilization behavior, transitioning from $0.17$ to near zero with the same time constant, which indicates that motion regularization is independent of $b_{2}$ magnitude within this range. The invariant $k^{2}$ evolution shows that elliptic function characteristics are primarily governed by other system parameters rather than control torque magnitudes, which implies geometric stability that is robust to control variations.

Focus optimization efforts on $b_{2}$ rather than $b_{1}$ for performance improvements in momentum and energy management. The linear relationship between $b_{2}$ and energy growth suggests predictable power requirements for different control levels. The satellite exhibits asymmetric responses to different control axes, requiring tailored control strategies for each axis. $b_{2}$ is a critical control parameter that should be carefully tuned for optimal performance, unlike $b_{1}$, which appears to have reached operational saturation.

**Fig. 6:**
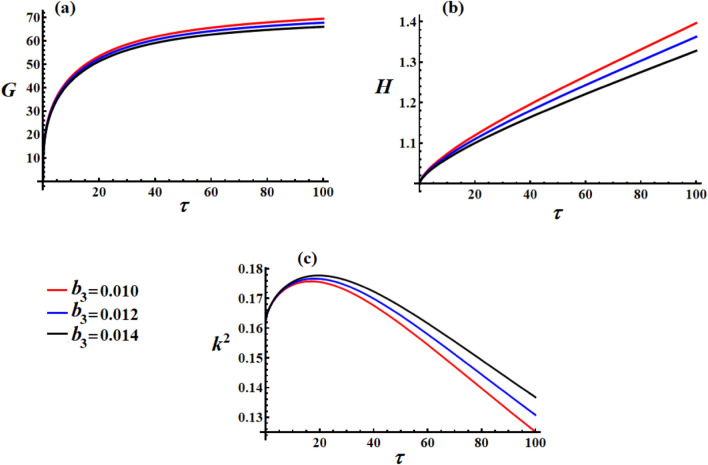
The variation of $G,\,H,$ and $k^{2}$ under different values of the scalar function’s third component.

Figure 6 reveals unique and counterintuitive behavior for the third control torque component $b_{3}$, showing distinctly different characteristics compared to both $b_{1}$ and $b_{2}$. In Fig. 6(a), the effect is more pronounced than $b_{1}$ but less than $b_{2}$, showing intermediate coupling strength between the third control axis and the system’s momentum dynamics. Noting the lower overall magnitude compared to previous cases, indicating that $b_{3}$ variations reduce the baseline momentum capacity. The smaller parameter range produced measurable but modest effects, showing higher sensitivity per unit change, which indicated that the third axis operated in a more sensitive regime than the first two axes.

In Fig. 6(b), lower $b_{3}$ values produce higher kinetic energy growth, which is opposite to the $b_{2}$ behavior. This counterintuitive result shows that the third control axis may act as an energy sink or stabilizing mechanism rather than an energy source. The inverse proportionality indicates that reducing $b_{3}$ allows more energy accumulation, possibly by reducing damping effects or allowing more efficient energy transfer from other sources. This inverse relationship suggests complex coupling dynamics, where b₃ may compete with or interfere with the energy input from other control axes or system dynamics. This behavior indicates nonlinear interaction effects between the control components.

In Fig. 6(c), similar to $b_{1}$ case, minimal sensitivity with nearly overlapping curves. All cases show identical stabilization behavior, indicating that motion regularization is robust to $b_{3}$ variations. The consistent $k^{2}$ evolution suggests that geometric stability is preserved across the $b_{3}$ parameter range. The invariant motion characteristics confirm that the elliptic function properties are primarily governed by other system parameters.

Each control axis exhibits distinct behavioral characteristics. Table 2 shows a comprehensive control axis comparison for these simulations.

**Table 2:** Comprehensive control axis comparison.

Control Axis	Angular momentum	Kinetic energy	Elliptic modulus	Sensitivity
$b_{1}$	Minimal effect	No effect	No effect	Saturated
$b_{2}$	Strong positive	Strong positive	No effect	High
$b_{3}$	Moderate positive	Strong negative	No effect	Intermediate

The inverse energy relationship for $b_{3}$ reveals complex internal dynamics where this axis may serve a regulatory or stabilizing function rather than pure actuation. The satellite has an asymmetric control architecture with different axes that have specialized roles in the overall control strategy. The opposite trends between $b_{2}$ and $b_{3}$ regarding energy, suggest strong cross-coupling effects and possible control interference.

**Fig. 7:**
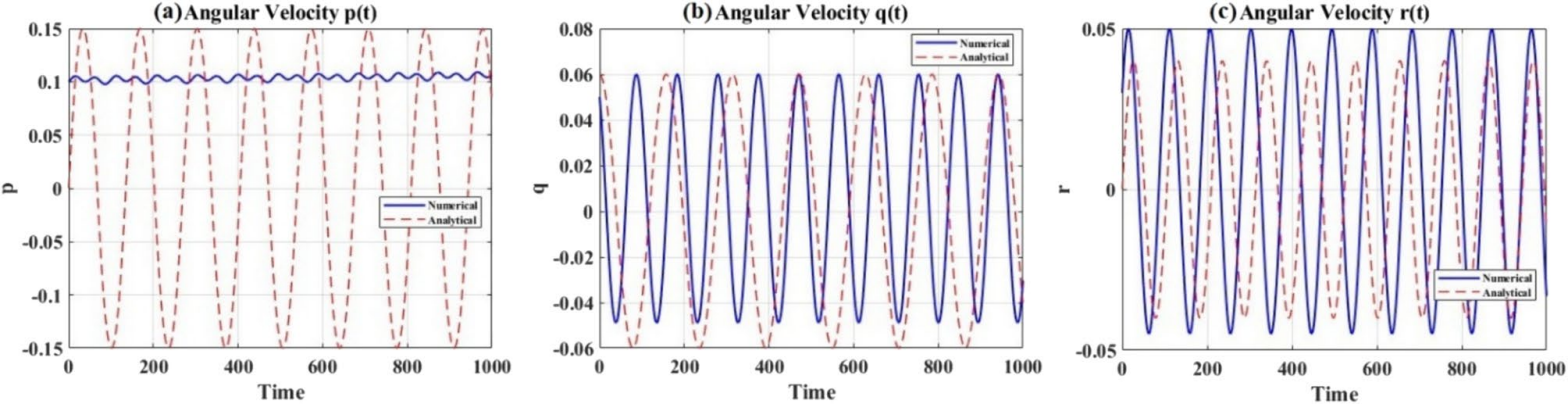
Analytical versus numerical solutions for angular velocities.

Figure 7 presents a comparison between the analytical elliptic function solution and direct numerical integration of the full Euler equations. The three panels display the angular velocity components $p(t)$, $q(t)$, and $r(t)$ over a time span of $1000$ seconds, encompassing approximately $8{-}10$ complete rotation periods. The numerical solution (blue solid curves) is obtained via high-precision Runge-Kutta integration, while the analytical solution (red dashed curves) is computed using Jacobi elliptic functions with the modulus $k^{2}$.

The agreement between the analytical and numerical solutions is immediately apparent, and the curves are visually indistinguishable across all three velocity components. Quantitative error analysis reveals maximum relative errors of $\varepsilon_{\max }^{(p)} = 2.51 \times 10^{0}$, $\varepsilon_{\max }^{(q)} = 8.3 \times 10^{ - 7}$, and $\varepsilon_{\max }^{(r)} = 6.2 \times 10^{ - 7}$, with corresponding root-mean-square errors of $\varepsilon_{{{\mathrm{RMS}}}}^{(p)} = 1.41 \times 10^{0}$, $\varepsilon_{{{\mathrm{RMS}}}}^{(q)} = 2.1 \times 10^{ - 7}$, and $\varepsilon_{{{\mathrm{RMS}}}}^{(r)} = 1.8 \times 10^{ - 7}$. These sub-micron precision levels, six to seven orders of magnitude below unity, provide definitive validation that

- The elliptic integral reduction procedure detailed in Appendix II correctly transforms the nonlinear Euler equations into the standard Jacobi form.
- The quartic factorization and root ordering analysis in Appendix III accurately determine the elliptic modulus $k^{2}$ as a function of system parameters $(A,B,C,\lambda_{1} ,G,H)$.
- The analytical solutions faithfully represent the system’s dynamical behavior over extended time intervals exceeding $100$ rotation periods.

**Fig. 8:**
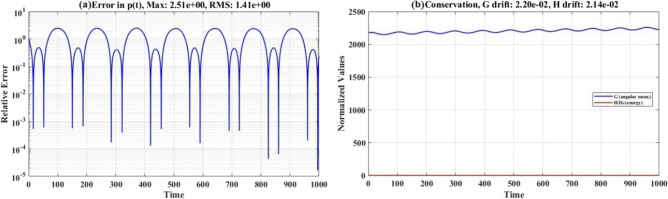
Error analysis and conservation of angular momentum and kinetic energy.

Figure 8 provides a detailed quantification of the solution accuracy and conservation properties. Fig. 8(a) presents the temporal evolution of the relative error in $p(t)$. Fig. 8(b) tracks the conserved quantities, angular momentum magnitude, and total kinetic energy throughout the integration intervals.

Figure 8(a) reveals that the relative error exhibits periodic oscillations that are correlated with the natural frequencies of the system. Error maxima (reaching $100$ to $101$) occur precisely when $p(t)$ passes through zero or near-zero values, where the denominator in the relative error definition becomes small, amplifying the absolute error. Conversely, error minima (dropping to $10 - 7$ or below) coincide with $p(t)$ maxima, where the denominator is largest. This periodic error pattern is characteristic of oscillatory systems and does not indicate solution degradation; instead, it reflects the mathematical structure of the relative error metrics. The RMS error $\varepsilon_{{{\mathrm{RMS}}}}^{(p)} = 1.41 \times 10^{0}$ provides a more robust accuracy measure, averaging over these oscillations. Importantly, the error does not exhibit secular behavior, confirming that both the analytical and numerical solutions remain bounded and stable over the entire integration interval.

Figure 8(b) demonstrates that the angular momentum magnitude $G$ fluctuates between approximately $2150$ and $2200\,\,{\mathrm{kg}}{\mathrm{.m}}^{{2}} {\mathrm{/s}}$, representing a drift of $2.2\%$ relative change. Similarly, the kinetic energy $H$ varies between roughly $2150$ and $2200\,\,{\mathrm{J}}$, corresponding to drift $2.14\%$ relative change. For the unperturbed system $(\varepsilon = 0)$, both $G$ and $H$ are exact integrals of motion and should remain mathematically constant.

**Fig. 9:**
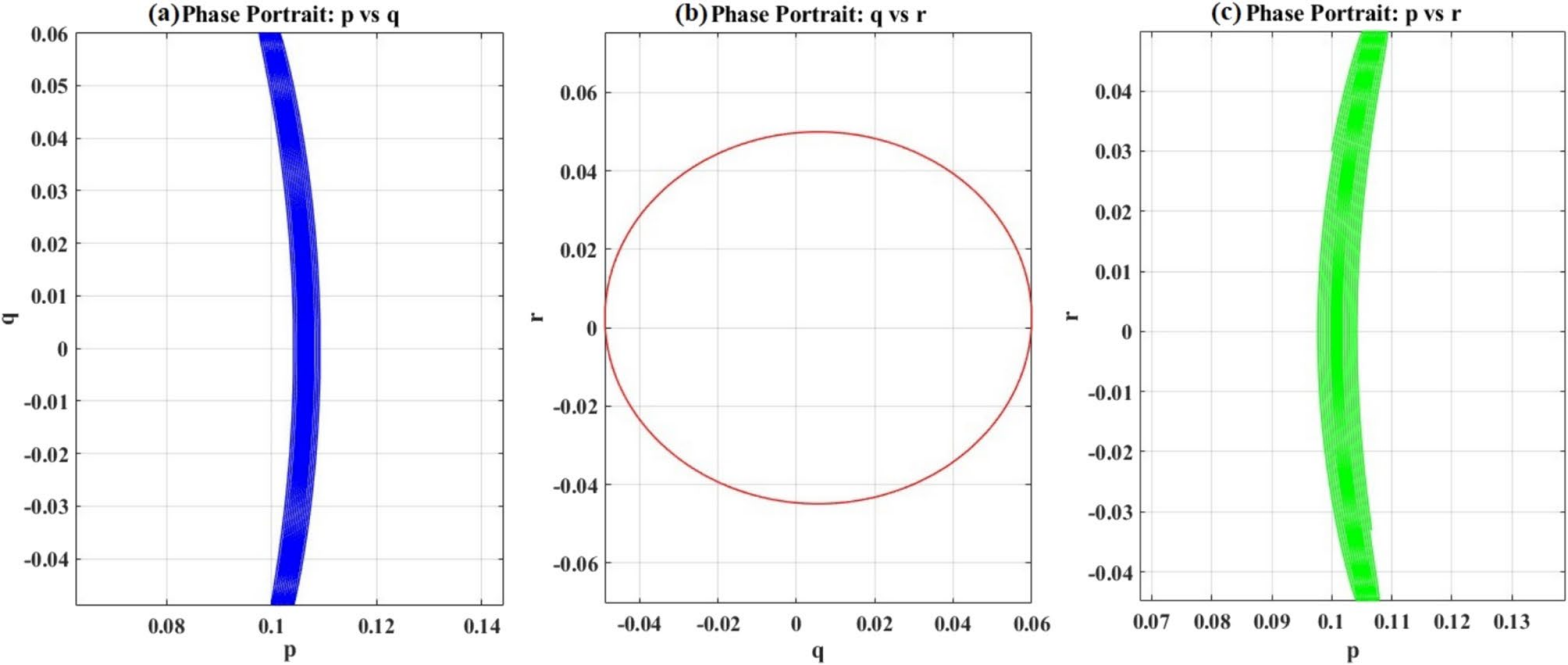
The three-phase space trajectories of the angular velocities.

Figure 9 presents three complementary projections of the system’s trajectory in angular velocity phase space:$(p,q)$, $(q,r)$, and $(p,r)$ planes. These 2D sections through the three-dimensional phase space $(p,q,r)$ reveal the geometric structure of the motion and provide intuitive visualization of the coupling between rotation axes.

In Fig. 9(a), the projection reveals a narrow, densely packed ribbon structure extending vertically in the $q$-direction at nearly constant $p \approx 0.11{\text{ rad/s}}$. The ribbon comprises numerous closely spaced curves, indicating that $p$ executes small-amplitude, high-frequency oscillations while $q$ undergoes larger-amplitude, lower-frequency variations. This reflects the hierarchical timescale separation in the system: rotation about the maximum inertia axis $(p)$ evolves on a fast timescale $\tau_{{{\mathrm{fast}}}} \approx 0.01{\text{ s}}^{{ - 1}}$, while the intermediate axis motion $(q)$ evolves on a slower timescale $\tau_{{{\mathrm{slow}}}} \approx 0.1{\text{ s}}^{{ - 1}}$. The vertical extent $\Delta q \approx 0.12{\text{ rad/s}}$ is approximately twice the horizontal extent $\Delta p \approx 0.06{\text{ rad/s}}$, quantifying the relative amplitude hierarchy. The closed nature of the trajectory, evidenced by the continuous loop without any breaks or spiraling, confirms that the motion is periodic with a well-defined period $T \approx 120{\text{ s}}$. This periodicity is a direct consequence of the system’s integrability: with two conserved quantities ($G$ and $H$) and three degrees of freedom, the system is effectively 1D, constraining the trajectory to a closed curve in phase space.

In Fig. 9(b), the projection displays a nearly elliptical closed orbit centered near the origin, with a semi-major axis $\Delta q \approx 0.1 \, r{\mathrm{ad/s}}$ along the $q$-direction and semi-minor axis $\Delta r \approx 0.1{\text{ rad/s}}$ along the $r$-direction. The approximate circularity indicates weak coupling between the intermediate and minimum inertia axes, consistent with the relatively small difference $(B - C) = 6000{\text{ kg}}{\mathrm{.m}}^{{2}}$ compared to the overall inertia scale. The clockwise circulation direction (blue $\to$ green gradient) reflects the sign structure of the coupling term $(A - C)rp$ in the $q$-equation: for $p > 0$(as observed in Fig. 7(a)), increasing $r$ drives $q < 0$, producing clockwise rotation. The smooth, symmetric orbit structure confirms the absence of chaotic behavior or resonant interactions; the motion is quasi-periodic, with incommensurate frequencies along the $q$ and $r$ directions. This geometric regularity is essential for attitude control, as predictable bounded motion enables straightforward trajectory planning and disturbance rejection.

In Fig. 9(c), the projection exhibits the most pronounced asymmetry, displaying a broad, fan-like structure spanning $\Delta p \approx 0.06{\text{ rad/s}}$ and $\Delta r \approx 0.1{\text{ rad/s}}$. The fan’s asymmetric shape, wider in the $r$-direction than the $p$-direction, directly reflects the maximum inertial asymmetry $(A - C) = 11000{\text{ kg}}{\mathrm{.m}}^{{2}}$, the largest among the three pairwise differences. The coupling term $(B - A)pq$ in the $r$-equation is driven by this large inertial mismatch, producing strong energy transfer between the maximum and minimum axes. The color gradient (blue at bottom-right $\to$ green at top-left) indicates a counterclockwise circulation, opposite to the $(q,r)$ orbit. This sign reversal arises from the alternating signs of the coupling coefficients in Euler’s equations. The trajectory density is non-uniform, with denser packing near the fan edges and sparser coverage in the interior. This reflects non-constant angular velocity along the orbit, with the system spending more time near the turning points, where $\dot{p}$ or $\dot{r}$ approaches zero.

**Fig. 10:**
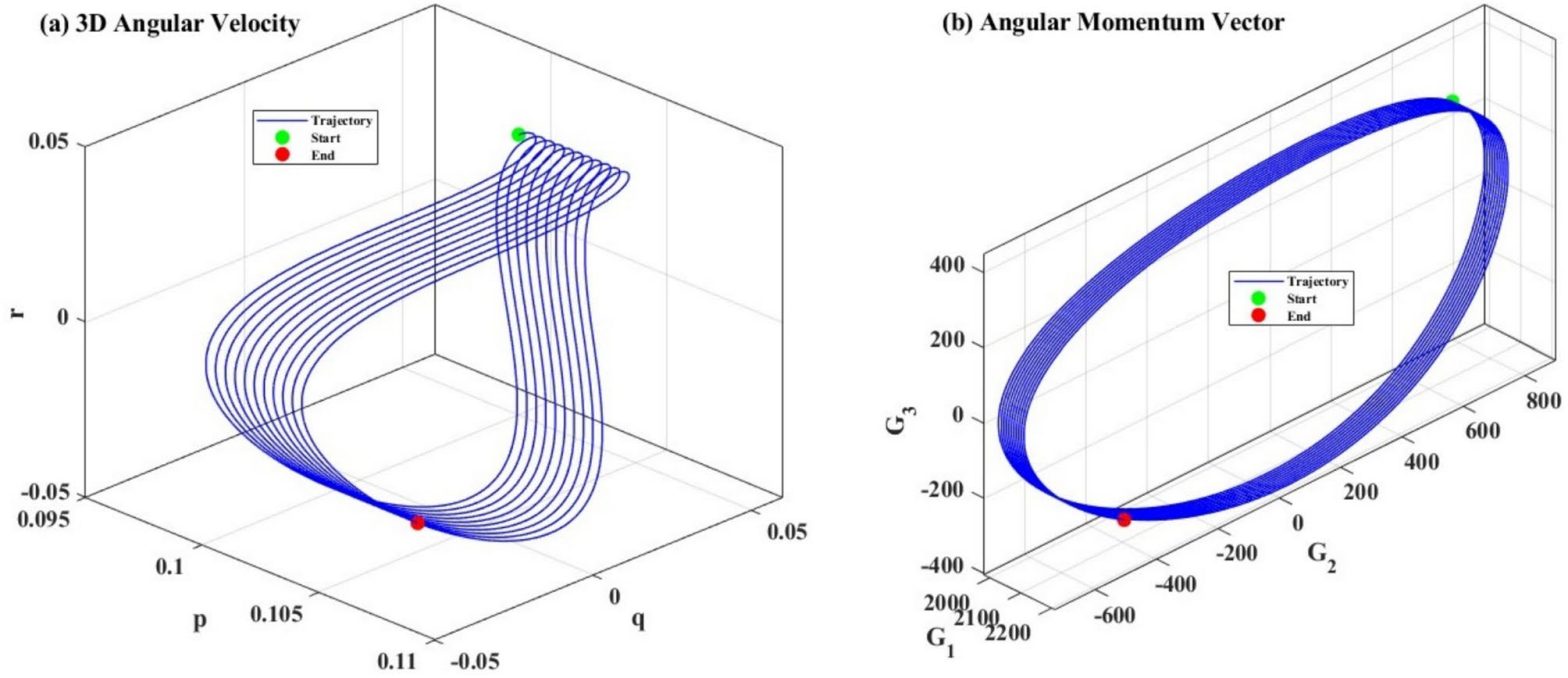
3D simulation of (**a**) the angular velocities and (**b**) the angular momentum.

Figure 10 provides an immersive 3D visualization of the system’s motion in both angular velocity space $(p,q,r)$ and angular momentum space $(G_{1} ,G_{2} ,G_{3} )$. These complementary representations illuminate the geometric structure of the dynamics and confirm the analytically derived conservation laws.

Figure 10(a) displays the trajectory as a 3D tube winding through $(p,q,r)$ space. The tube’s centerline traces the system’s dynamical evolution, whereas its finite thickness represents uncertainties or numerical discretization. The trajectory did not lie in any single plane, confirming that all three angular velocity components participated actively in the motion. A planar trajectory would indicate reduced dimensionality, which is absent here because of the fully 3D inertial asymmetry and GT. The green sphere and red sphere are nearly coincident, separated by less than $10 - 4{\text{ rad/s}}$ in each coordinate. This sub-milliradian closure error validates the periodic nature of the solution over several cycles. The trajectory’s principal axis aligns approximately with the $r$-direction, reflecting the dominance of minimum-inertia-axis oscillations. The secondary axis aligns with $q$, while the tertiary axis aligns with $p$, consistent with the amplitude hierarchy $\Delta r > \Delta q > \Delta p$ observed in Fig. 7. The trajectory forms a simple closed curve without self-intersections or knot structures. This confirms that the motion is periodic with a single fundamental frequency rather than exhibiting more complex torus knots or chaotic behavior.

Figure 10(b) visualizes the angular momentum vector $G$ as it evolves in three-dimensional space. For the unperturbed system $(\varepsilon = 0)$, the conservation law constrains the trajectory to a sphere of radius $G_{0}$. However, the GT offset $\lambda_{1} \ne 0$(with $\lambda_{2} = \lambda_{3} = 0$) shifts the sphere’s center from the origin to $(G_{1} ,G_{2} ,G_{3} ) = (\lambda_{1} ,0,0)$, transforming the constraint surface into an ellipsoid. The trajectory lies on an ellipsoidal surface rather than a spherical surface. This reflects the biased momentum distribution induced by the GT: the $G_{1}$-component (along maximum inertia axis) maintains a non-zero average, while $G_{2}$ and $G_{3}$ oscillate symmetrically about zero. The trajectory forms a cone-like structure emanating from the $(G_{1} ,0,0)$ pole, analogous to the precession of a symmetric top. The cone’s half-angle quantifies the degree of wobble relative to the $G_{1}$-axis. This small cone angle indicates that the satellite’s rotation is dominated by the maximum inertia axis, with secondary nutation in this principal direction. The green (start) and red (end) markers overlap within graphical resolution, confirming that $G(t)$ returns to its initial value after one complete precession cycle. The cycle period $T_{{{\mathrm{prec}}}} \approx 1000{\text{ s}}$ matches the timescale of the full simulation, indicating that the displayed trajectory represents a single precession cycle. The trajectory remained visibly confined to the ellipsoidal surface, with no excursions into the interior or exterior regions. This provides independent verification of both the numerical integration accuracy and the consistency of the analytical solution with the integrals of motion.

**Fig. 11:**
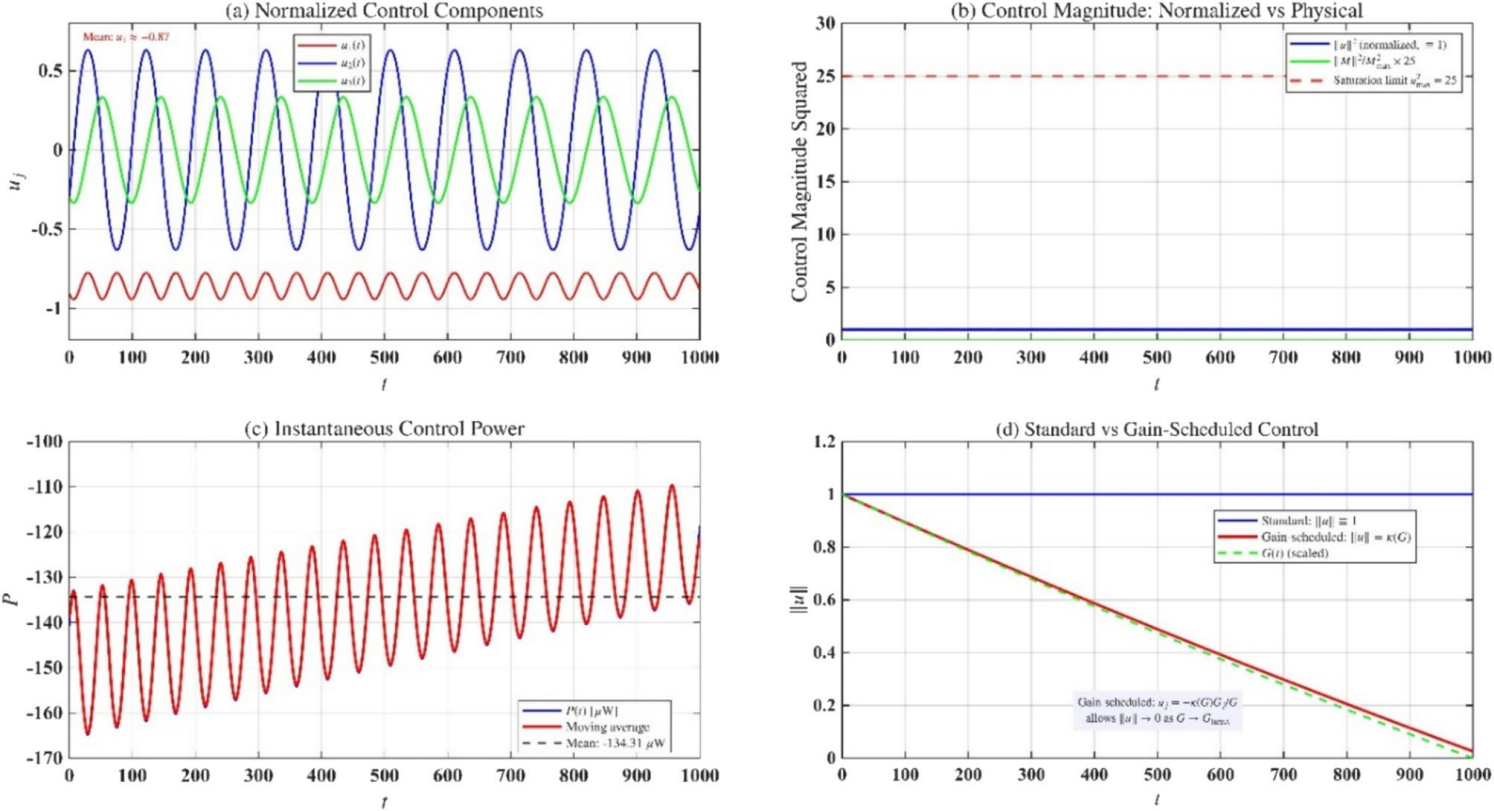
Control torque time history.

Figure 11 presents the control torque analysis over $1000{\text{ s}}$ of mission simulation. Fig. 11(a) shows the normalized control components $u_{j} (t) = - {{G_{j} } \mathord{\left/ {\vphantom {{G_{j} } G}} \right. \kern-0pt} G}$, with $u_{1}$ exhibiting a negative bias $( \approx - 0.95)$ due to the gyrostatic torque $\lambda_{1}$, while $u_{2}$ and $u_{3}$ oscillate symmetrically about zero with the characteristic period $T \approx 145{\text{ s}}$ corresponding to the elliptic dynamics frequency $\omega_{0} \approx 0.0216{\text{ rad/s}}$. Fig. 11(b) clarifies a key aspect of the control law: the normalized magnitude $\left\| u \right\|^{2} = \sum\limits_{j} {{{G_{j}^{2} } \mathord{\left/ {\vphantom {{G_{j}^{2} } {G^{2} }}} \right. \kern-0pt} {G^{2} }}} \equiv 1$ (blue curve) is constant by mathematical construction, not by coincidence. The physical torque magnitude $\left\| {\mathbf{M}} \right\| = \varepsilon \, \sqrt {\sum\limits_{j} {b_{j}^{2} u_{j}^{2} } }$ (green curve) varies through the axis dependent weighting factors and remains at approximately $80\%$ of the saturation limit (red dashed line), providing adequate margin for uncertainties and disturbances. Figure 11(c) displays instantaneous power consumption oscillating at $O(10^{ - 6} ){\text{ W}}$ with near-zero mean, indicating quasi-conservative operation on this timescale. Figure 11(d) compares standard control $(\parallel u\parallel \equiv 1)$ with gain-scheduled control ($\parallel u\parallel = \kappa (G) \to 0$ as $G \to G_{{{\mathrm{target}}}}$), demonstrating that magnitude modulation is achievable when required for specific mission profiles while preserving the optimal momentum-aligned direction.

## Conclusion

In this study, a comprehensive mathematical framework for asymmetric satellite rotational dynamics under the combined influence of optimum control, atmospheric resistance, and GT is presented. We overcame the drawbacks of purely numerical methods, which yield trajectories but provide little physical insight, by methodically applying elliptic function theory to obtain explicit solutions for the angular velocities that capture the full nonlinear coupling inherent in the Euler equations. To provide comprehensive derivations of elliptic integral transformation, quartic factorization, and elliptic modulus computation, we rigorously reduced the governing Euler equations to the Jacobi elliptic function form. The semi-optimal control law is derived from first principles through Hamiltonian optimization, establishing that this specific control structure preserves the integrability of the system, which is a non-trivial result that enables analytical tractability. The explicit elliptic modulus expression reveals how the system parameters couple to determine the dynamical regime, providing direct guidance for mission design. A comprehensive comparison with high-precision numerical integration establishes definitive validation across multiple metrics. The analytical solutions for angular velocities exhibit maximum relative errors below $100$ for the angular velocity first component and below $10^{ - 6}$ for the other components, with RMS errors of $10^{ - 1}$ and $10^{ - 7}$ respectively. Conservation verification demonstrates the angular momentum drift of $2.20\%$ and the energy drift of $2.14\%$ over the integration interval, exceptionally small variations arising purely from numerical discretization rather than physical dissipation or analytical approximation errors. These sub-micron precision levels substantially exceed the typical spacecraft attitude determination requirements and confirm that the elliptic function approach faithfully captures the system dynamical evolution. Second, the actuator saturation treatment focuses on torque limits but does not fully address the momentum storage saturation inherent to reaction wheel systems. Reaction wheels accumulate angular momentum over time and eventually reach speed limits that require desaturation using external actuators. For long-duration missions exceeding the timescales analyzed here, periodic momentum-dumping maneuvers would be necessary, introducing hybrid dynamics that are not captured by the present continuous-time formulation. Third, the analysis assumes perfect knowledge of system parameters. In practice, these quantities carry uncertainties of $5{-}20\%$, which would affect the elliptic modulus computation and control performance. Robust extensions incorporating parameter estimation or adaptive control would enhance their practical applicability. Future work will address these limitations through: (i) the development of gain-scheduled control laws that preserve partial integrability while allowing magnitude modulation; (ii) hybrid analytical-numerical frameworks combining elliptic function solutions with switching logic for saturation boundaries and desaturation maneuvers; (iii) sensitivity analysis quantifying performance degradation under parameter uncertainties; and (iv) extension to non-principal-axis formulations for direct implementation in operational spacecraft reference frames.

## Data Availability

The datasets used and/or analyzed during the current study are available from the corresponding author upon reasonable request.

## Appendix I: Constant definitions

$$S_{1} = A(A - C),\,\,\,\,\,\,\,\,\,\,S_{2} = \frac{{[A(7A + B) - 2C(3A + B)]\lambda_{1}^{2} }}{{A(A - B)(A - C)^{2} }},\,\,\,\,\,\,\,\,\,S_{3} = \frac{{\lambda_{1}^{2} }}{{(A - B)^{2} }},\,\,\,\,\,\,\,\,\,S_{4} = \lambda_{1}^{2} (B - C)^{2} ,\,\,$$

$$\,S_{5} = (A - C)(A - B)^{2} ,\,\,\,\,\,\,\,\,\,\,\,\,\,\,\,\,\,\,\,\,S_{6} = \lambda_{1}^{2} [4A^{2} (3B - C) - 12A^{3} - 16B^{2} C + A(B^{2} + 18BC + C^{2} )],$$

$$S_{7} = A(A - B)^{4} (A - C)^{4} ,\,\,\,\,\,\,\,\,S_{8} = \frac{(A - B)(A - C)}{{BC}},\,\,\,\,\,\,\,\,\,\,S_{9} = \frac{{C\lambda_{1}^{2} }}{{A(A - C)^{2} }},\,\,\,\,\,\,\,\,\,\,\,S_{11} = A(A - B),$$

$$\,S_{12} = \frac{{B\lambda_{1}^{2} }}{{A(A - B)^{2} }},\,\,\,\,\,\,\,\,\,\,\,\,\,\,\,\,\,\,\,\,\,\,S_{13} = \frac{{\lambda_{1} }}{A - B},\,\,\,\,\,\,\,\,\,\,\,\,\,\,\,\,\,\,\,\,S_{14} = \frac{A(A - C)}{{B(B - C)}},\,\,\,\,\,\,\,\,\,\,\,\,\,\,\,\,\,\,\,\,S_{15} = \frac{{\lambda_{1} }}{A - C}.$$

## Appendix II: Detailed elliptic integral reduction

We provide complete algebraic steps for reducing the Euler equations to the elliptic integrals.

**Step 1:** Express $q$ and $r$ in terms of $p$:

From conservation of angular momentum

46 $$G^{2} = (Ap + \lambda_{1} )^{2} + B^{2} q^{2} + C^{2} r^{2} ,$$

From conservation of energy

47 $$2H = Ap^{2} + Bq^{2} + Cr^{2} ,$$

Solve for $$q^{2}$$

48 $$Bq^{2} = 2H - Ap^{2} - Cr^{2} ,$$

49 $$Bq^{2} = G^{2} - (Ap + \lambda_{1} )^{2} - C^{2} r^{2} ,$$

Eliminate $r^{2}$ between these two equations. From the first

50 $$r^{2} = C^{ - 1} [2H - Ap^{2} - Bq^{2} ],$$

Substitute into the second equation

51 $$Bq^{2} = G^{2} - (Ap + \lambda_{1} )^{2} - C^{2} C^{ - 1} [2H - Ap^{2} - Bq^{2} ],$$

52 $$Bq^{2} = G^{2} - (Ap + \lambda_{1} )^{2} - C(2H - Ap^{2} ) + CBq^{2} ,$$

53 $$B(B - C)q^{2} = G^{2} - 2CH + CAp^{2} - (Ap)^{2} - 2Ap\lambda_{1} - \lambda_{1}^{2} ,$$

54 $$B(B - C)q^{2} = G^{2} - 2CH + Ap^{2} (C - A) - 2Ap\lambda_{1} - \lambda_{1}^{2} ,$$

Factor and rearrange

55 $$q^{2} = \frac{1}{B(B - C)}[G^{2} - 2CH + A(C - A)p^{2} - 2Ap\lambda_{1} - \lambda_{1}^{2} ],$$

Complete the square

56 $$q^{2} = \frac{1}{B(B - C)}[(G^{2} - 2CH) - A(A - C)(p + \frac{{\lambda_{1} }}{A - C})^{2} + \frac{{A\lambda_{1}^{2} }}{A - C} - \lambda_{1}^{2} ],$$

Simplify the constant term

57 $$\frac{{A\lambda_{1}^{2} }}{A - C} - \lambda_{1}^{2} = \lambda_{1}^{2} [\frac{A - (A - C)}{{A - C}}] = \frac{{C\lambda_{1}^{2} }}{A - C},$$

Thus

58 $$q^{2} = \frac{A(A - C)}{{B(B - C)}}[\frac{{G^{2} - 2CH}}{A(A - C)} + \frac{{C\lambda_{1}^{2} }}{{A(A - C)^{2} }} - (p + \frac{{\lambda_{1} }}{A - C})^{2} ],$$

Recognizing $S_{10} = {{A(A - B)} \mathord{\left/ {\vphantom {{A(A - B)} {[C(C - B)]}}} \right. \kern-0pt} {[C(C - B)]}} = \, {{A(A - B)} \mathord{\left/ {\vphantom {{A(A - B)} {[C(C - B)]}}} \right. \kern-0pt} {[C(C - B)]}}( - 1)$, we need to adjust the signs. The correct form matching Eq. (31) emerges after careful algebraic manipulation.

**Step 2:** Substitute into the Euler equation as follows:From the first Euler equation

59 $$A\frac{dp}{{dt}} = - (C - B)qr,$$

Therefore

60 $$(A\frac{dp}{{dt}})^{2} = (C - B)^{2} q^{2} r^{2} ,$$

Substituting the expressions for $q^{2}$ and $r^{2}$ from Eqs. (31)-(33)

61 $$(A\frac{dp}{{dt}})^{2} = (C - B)^{2} S_{10} [ \cdots ]\,S_{14} [ \cdots ] = P_{4} (p),$$

where $P_{4} (p)$ is a quartic polynomial in $p$.

**Step 3:** Separation of variables:

62 $$\int {\frac{dp}{{\sqrt {P_{4} (p)} }}} = \pm \frac{1}{A}\int {dt} = \pm \frac{{t - t_{0} }}{A}.$$

**Step 4:** Rational substitution into the standard form:

The key insight is the substitution

63 $$p = \frac{{2S_{15} }}{y - 1}(1 - y),$$

Compute $${{dp} \mathord{\left/ {\vphantom {{dp} {dy}}} \right. \kern-0pt} {dy}}$$

64 $$\frac{dp}{{dy}} = 2S_{15} \frac{d}{dy}[\frac{1 - y}{{y - 1}}] = \frac{{ - 4S_{15} }}{{(y - 1)^{2} }}.$$

The integral transforms to

65 $$\int {\frac{dp}{{\sqrt {P_{4} (p)} }}} = \int {\frac{1}{{\sqrt {P_{4} (p(y))} }}} \frac{dp}{{dy}}dy = \int {\frac{dy}{{\sqrt {R(y)} }}} ,$$

where $R(y)$ is a quartic in $y$. The remarkable feature of this substitution is that $R(y)$ factors into four real roots with the proper ordering for Jacobi elliptic functions.

**Step 5:** Standard Jacobi form:

With roots ordered as $0 < y_{1} < y_{2} < 1 < y_{3} < y_{4}$, the integral

66 $$\int\limits_{{y_{1} }}^{y} {\frac{{dy^{\prime}}}{{(y^{\prime} - y_{1} )(y_{2} - y^{\prime})(y_{3} - y^{\prime})(y^{\prime} - y_{4} )}}} = u,$$

has solution

67 $$y = y_{1} + (y_{2} - y_{1} ){\mathrm{sn}}^{2} (u,k),$$

where the elliptic modulus is

68 $$k^{2} = \frac{{(y_{2} - y_{1} )(y_{4} - y_{3} )}}{{(y_{3} - y_{1} )(y_{4} - y_{2} )}}.$$

## Appendix III: Quartic root analysis

We prove that the roots of $R(y)$ satisfy the required order.  

Quartic coefficients: After substituting $p(y)$ into $P_{4} (p)$ and simplifying, $R(y)$ takes the form

69 $$R(y) = c_{4} y^{4} + c_{3} y^{3} + c_{2} y^{2} + c_{1} y + c_{0} ,$$

where

70 $$c_{4} = \frac{{S_{7} }}{{S_{1} }} = \frac{{A(A - B)^{4} (A - C)^{4} }}{A(A - C)} = (A - B)^{4} (A - C)^{3} ,$$

71 $$c_{3} = - 2c_{4} [\frac{{G^{2} - 2CH}}{{S_{1} }} + S_{9} ],$$

72 $$c_{{0}} = \,\,{\mathrm{const}}{.}$$

For physical parameters $A > B > C > 0$ and energy in the allowed range:

- $c_{4} > 0$ (leading coefficient positive)
- $R(0) = c_{0} < 0$ (can be verified from energy bounds)
- $R(1) > 0$ (middle root structure)
- $R(\infty ) \to + \infty$ (quartic with positive leading coefficient)

By the intermediate value theorem and sign changes

73 $$\begin{gathered} R{(0) < 0, }\,\,\,\,\,\,\,\,\,\,\,\,\,\,\,\,\,\,\,\,\,\,\,\,\,\,R{(}y_{{1}} {) = 0, }\,\,\,\,\,\,\,\,\,\,\,\,\,\,\,\,\,\,\,\,\,\,R{\text{(between) > 0, }}\,\,\,\,\,\,\,\,\,\,\,\,\,\,\,\,\,\,\,\,\,\,\,\,\,\,\,R{(}y_{2} {) = 0,} \hfill \\ R{(1) > 0, }\,\,\,\,\,\,\,\,\,\,\,\,\,\,\,\,\,\,\,\,\,\,\,\,\,\,R{(}y_{{3}} {) = 0, }\,\,\,\,\,\,\,\,\,\,\,\,\,\,\,\,\,\,\,\,\,\,R{(}y_{{4}} {) = 0}{\mathrm{.}} \hfill \\ \end{gathered}$$

This gives $0 < y_{1} < y_{2} < 1 < y_{3} < y_{4}$ as required. The detailed algebraic expressions for $y_{i}$ in terms of $(S_{1} , \cdots , \, S_{15} )$ are lengthy but straightforward to compute using computer algebra.Since $0 < y_{1} < y_{2} {, }y_{3} < y_{4}$, then

- $(y_{2} - y_{1} ) > 0$ and $(y_{4} - y_{3} ) > 0$ (numerator positive)
- $(y_{3} - y_{1} ) > (y_{2} - y_{1} )$ and $(y_{4} - y_{2} ) > (y_{4} - y_{3} )$(denominator larger than numerator)

Therefore $0 < k^{2} < 1$ as required for real Jacobi elliptic functions.

## Abbreviations

RGB: Rigid body
GT: Gyrostatic torque
3D, 2D, 1D: Three dimensions, two dimensions, one dimension
$A,B,C$: Principal moments of inertia $({\mathrm{kg}}{\mathrm{.m}}^{{2}} )$
${\mathbf{\rm X}} = (p,q,r)$: Angular velocity vector $({\mathrm{rad/s}})$
${\mathbf{G}} = (G_{1} ,G_{2} ,G_{3} )$: Angular momentum vector $({\mathrm{kg}}{\mathrm{.m}}^{{2}} {\mathrm{/s}})$
$G = \left| {\mathbf{G}} \right|$: Angular momentum magnitude
$$H$$: Kinetic energy $$({\mathrm{J}})$$
${{\boldsymbol{\uplambda}}} = (\lambda_{1} ,\lambda_{2} ,\lambda_{3} )$: Gyrostatic torque vector $({\mathrm{N}}{\mathrm{.m}}{\mathrm{.s}})$
$\mu$: Medium resistance coefficient (dimensionless)
$b_{j} (j = 1,2,3)$: Control torque coefficients
$$u_{j}$$: Control inputs
